# Nutritional regulation of the tumor immune microenvironment: metabolic mechanisms, immunotherapy response, and nursing implications

**DOI:** 10.3389/fimmu.2026.1938564

**Published:** 2026-10-12

**Authors:** Danwei Zhong, Congjue Li, Zunyao Xiao

**Affiliations:** 1 Department of Gastrointestinal Oncology II, The Second Hospital of Dalian Medical University, Dalian, Liaoning, China; 2 Department of Orthopedics, The Second Hospital of Dalian Medical University(Diamond Bay Campus), Dalian, Liaoning, China; 3 Department of Gynecology II, The Second Hospital of Dalian Medical University, Dalian, Liaoning, China

**Keywords:** cancer immunotherapy, gut microbiota, nutritional regulation, oncology nursing, tumor immune microenvironment

## Abstract

Nutrition is increasingly recognized as an active regulator of the tumor immune microenvironment rather than a passive supportive-care factor. Beyond maintaining body weight and treatment tolerance, dietary inputs and nutritional interventions can reshape systemic metabolism, gut microbial ecology, stromal barriers, immune-cell fitness, and response to immune checkpoint blockade. Recent experimental and translational studies indicate that fasting-mimicking diets, ketogenic interventions, carbohydrate-derived signals, amino acid availability, lipid mediators, artificial sweeteners, and microbiota-derived metabolites may either enhance or impair antitumor immunity depending on tumor type, host metabolic status, microbial composition, and treatment context. These effects involve multiple interconnected mechanisms, including CD8^+^ T-cell activation or exhaustion, myeloid-cell reprogramming, cancer-associated fibroblast-mediated immune exclusion, adipocyte and hepatocyte metabolic signaling, and microbiome-dependent immune modulation. Importantly, the same nutritional factor may produce beneficial, neutral, or harmful effects across different models, indicating that nutrition should not be translated into universal anticancer dietary prescriptions. This review summarizes current evidence linking nutritional regulation to tumor immune remodeling and immunotherapy response, with particular attention to nursing implications. Oncology nurses play a key role in nutritional screening, patient education, monitoring of extreme dietary practices, assessment of supplement and artificial sweetener use, and multidisciplinary nutritional management during immunotherapy.

## Highlights

- Nutrition actively reshapes tumor immunity through systemic metabolism, microbiota, and immune-cell fitness.
- Nutritional interventions may enhance or impair immunotherapy response depending on biological context.
- Nursing surveillance is essential to prevent unsafe diets and support precision nutrition during immunotherapy.

## 1 Introduction

Nutritional status has long been recognized as a major determinant of clinical outcomes in patients with cancer (1–5). Traditionally, cancer nutrition has focused on body weight loss, anorexia, sarcopenia, cachexia, protein–energy malnutrition, treatment tolerance, postoperative recovery, and quality of life. These aspects remain clinically important, particularly because malnutrition can reduce treatment adherence, increase toxicity, impair wound healing, and worsen survival. However, this classical view is no longer sufficient to explain the complex biological relationship between nutrition and cancer progression. Increasing evidence indicates that nutritional factors do not merely provide calories or substrates for tumor growth; they can actively reshape systemic metabolism, immune-cell function, gut microbial ecology, stromal behavior, and the tumor immune microenvironment (TIME) (6–9).

The TIME is metabolically constrained. Tumor cells, stromal cells, and infiltrating immune cells compete for glucose, amino acids, lipids, oxygen, and micronutrients within a spatially heterogeneous microenvironment. At the same time, immune cells require specific metabolic programs to maintain effector function. Activated CD8^+^ T cells depend on glycolysis, mitochondrial fitness, amino acid availability, and lipid metabolic balance to sustain proliferation, cytokine production, cytotoxic activity, and memory formation (10–14). Conversely, immunosuppressive cells such as myeloid-derived suppressor cells (MDSCs), tumor-associated macrophages (TAMs), regulatory T cells (Tregs), and cancer-associated fibroblasts (CAFs) can exploit metabolic stress to suppress antitumor immunity. Therefore, nutritional inputs may influence cancer immunity by modifying nutrient availability, metabolite signaling, immune-cell metabolic fitness, and stromal barriers.

Recent experimental and translational studies have shifted the field from a supportive-care perspective toward an immunometabolic perspective. For example, fasting-mimicking diet (FMD) has been shown to be feasible and safe in patients with cancer and to reshape systemic metabolism together with antitumor immune responses, suggesting that structured dietary interventions may influence immune competence rather than merely reduce caloric intake (15). In parallel, studies linking diet, gut microbiota, and immune checkpoint blockade (ICB) response have demonstrated that dietary patterns can influence microbial ecology and thereby shape immunotherapy outcomes (16). Importantly, not all nutritional exposures are beneficial. Sucralose consumption has been reported to impair cancer immunotherapy response through microbiome disruption, highlighting that common dietary additives may have clinically relevant immunologic consequences (17). These findings have important implications for oncology nursing and nutritional care. Patients receiving immunotherapy frequently ask whether they should fast, avoid sugar, adopt ketogenic diets, increase dietary fiber, consume probiotics, or use artificial sweeteners. Without mechanistic understanding, such questions may be answered with overly simplistic dietary advice. Nutritional regulation of the TIME is context dependent and shaped by tumor type, host metabolism, gut microbiota, treatment regimen, and nutritional risk. Therefore, this review considers nutrition as a modifiable immunometabolic variable that may influence immunotherapy response, toxicity, and nursing care.

Despite rapid progress, current discussions of nutrition and cancer immunity remain fragmented. Many studies focus on single nutrients or dietary strategies, whereas fewer integrate host metabolism, gut microbiota, metabolic organs, stromal remodeling, immune-cell function, and immunotherapy response into one translational framework (18–20). Moreover, evidence from *in vitro* studies, animal models, observational cohorts, early-phase clinical studies, and guideline-supported recommendations is often discussed without sufficient distinction. This creates a risk that mechanistic findings may be overinterpreted as clinical dietary advice. Therefore, this review aims to synthesize nutritional regulation of the tumor immune microenvironment through an evidence-stratified and cancer-context-dependent lens. We focus on how nutritional inputs are converted into metabolic mediators, how these mediators reshape immune and stromal cell states, and how these processes may influence immunotherapy response. We further discuss nursing-relevant considerations for nutritional screening, patient education, dietary behavior monitoring, and multidisciplinary referral, while emphasizing that most immunometabolic dietary strategies remain investigational rather than established clinical recommendations. Although several reviews have discussed diet, metabolism, the gut microbiome, and cancer immunity, the present review has a more specific scope. Rather than summarizing nutrition as a general supportive-care topic, we focus on how defined nutritional inputs and host metabolic states may remodel the tumor immune microenvironment and influence immunotherapy response. We also distinguish different levels of evidence, including *in vitro* mechanistic studies, preclinical models, observational cohorts, early-phase clinical studies, and guideline-supported recommendations. This approach allows us to separate mechanistic hypotheses from clinically actionable practices and to identify nursing-relevant assessment domains without implying that experimental dietary strategies are ready for routine clinical prescription.

## 2 Conceptual framework: from nutritional inputs to immune remodeling

Nutritional regulation of the TIME can be conceptualized as a stepwise but interconnected process. First, external nutritional inputs enter the host as dietary patterns, specific nutrients, dietary restriction strategies, supplements, or food additives. Second, these inputs are converted into metabolic mediators through host metabolism, microbial metabolism, or tumor-associated metabolic reprogramming. Third, these mediators act on tumor cells, immune cells, stromal cells, and metabolic organs. Finally, these cellular changes influence immune infiltration, immune suppression, antigen presentation, T-cell exhaustion, stromal exclusion, and response to immunotherapy. This framework provides a structure for understanding why the same dietary factor may have different effects in different tumor types or patient populations.

### 2.1 Nutritional inputs

The first layer of this framework consists of nutritional inputs. These include dietary interventions such as fasting, short-term starvation, FMD, ketogenic diets, and caloric restriction-like strategies. They also include specific macronutrient patterns, such as carbohydrate intake, fructose exposure, dietary fiber, amino acid availability, sulfur-containing nutrients, and lipid composition. In addition, non-nutritive but diet-associated exposures, such as artificial sweeteners, may also influence the TIME through microbiome-dependent or immune-metabolic mechanisms. FMD and short-term starvation represent metabolic stress interventions that may transiently reduce growth factor signaling, alter glucose and insulin availability, and promote immune remodeling. In clinical and experimental settings, FMD has been associated with systemic metabolic changes and enhanced antitumor immune responses (15, 21). Ketogenic diets represent another form of metabolic intervention, characterized by reduced carbohydrate intake and increased ketone-body production. Ketogenesis has been linked to suppression of immunosuppressive chemokine signaling in colorectal cancer and improved ICB response in prostate cancer models (22).

Carbohydrate-derived inputs are more complex than a simple “high sugar versus low sugar” model. Fructose, glucose, and galactose may influence antitumor immunity through different metabolic organs and immune pathways. For example, fructose-mediated adipocyte metabolism can support antitumor CD8^+^ T-cell responses, whereas high-glucose drink supplementation has been reported to modulate gut microbiota and enhance antitumor immunity in glioblastoma models (23). Galactose may reprogram hepatocyte-related immune regulation and prevent CD8^+^ T-cell exhaustion (24). In contrast, artificial sweeteners such as sucralose may impair ICB efficacy through microbiome disruption, underscoring the need to distinguish natural nutrients, metabolic substrates, and non-nutritive dietary additives (17). Amino acids and lipid-derived nutrients also serve as important immunometabolic regulators. Methionine restriction has been explored as an anticancer strategy, but sulfur deficiency induced by methionine restriction may impair antitumor immunity through gut microbiota-dependent mechanisms (25). Leucine availability may shape antigen-presenting neutrophil states and enhance anti-PD-1 response (26). Lipid-related inputs, including omega-3 fatty acids and cholesterol-associated metabolic pathways, may influence inflammation resolution, myeloid suppression, and ICB efficacy (27, 28). Collectively, these studies indicate that nutritional inputs should be evaluated not only by their caloric value, but also by their ability to regulate immune metabolism.

### 2.2 Metabolic mediators

The second layer of the framework involves metabolic mediators generated from nutritional inputs. These mediators include host-derived metabolites, microbiota-derived metabolites, lipid mediators, ketone bodies, sulfur metabolites, and tumor-associated metabolic intermediates. They serve as biochemical messengers that connect diet with immune regulation. β-hydroxybutyrate is one of the best-characterized ketone bodies linking nutritional intervention to immune remodeling. In ketogenic or ketogenesis-enhancing conditions, β-hydroxybutyrate may influence gene expression, inflammatory signaling, and stromal chemokine production. In colorectal cancer, ketogenesis attenuates KLF5-dependent CXCL12 production, thereby helping overcome an immunosuppressive microenvironment (22). In prostate cancer models, ketogenic diet-related metabolic changes can enhance immune checkpoint blockade response.

Microbiota-derived metabolites represent another critical class of mediators. Diet-driven changes in microbial ecology can alter the production of bioactive metabolites that influence dendritic cells, T cells, myeloid cells, and systemic inflammatory tone. Trimethylamine N-oxide (TMAO), commonly discussed in cardiovascular metabolism, has been shown to drive immune activation and improve response to ICB in pancreatic cancer models (29). Other microbial or microbiota-linked metabolites, including flavin adenine dinucleotide (FAD)-related pathways, may mobilize adipocyte lipid metabolism and promote antitumor immunity (30). These findings suggest that the immunologic effects of microbial metabolites are disease-context dependent and cannot be interpreted solely through non-cancer metabolic paradigms. Sulfur metabolism is also emerging as an important mediator of nutritional immunology. Hydrogen sulfide (H_2_S) can shape a favorable immune microenvironment through the PPARγ/miR-21/PTEN pathway in colon cancer (31). In contrast, methionine restriction-induced sulfur deficiency can impair antitumor immunity through gut microbiota-dependent mechanisms (25). These apparently divergent observations suggest that sulfur-related pathways require careful interpretation, because both excess and deficiency may have distinct effects depending on the tumor model, microbial context, and immune-cell state.

Lipid mediators provide another bridge between nutrition and TIME remodeling. Omega-3 fatty acid metabolism and soluble epoxide hydrolase inhibition can enhance cancer immunotherapy by modulating lipid-derived inflammatory signals (27). In metabolic dysfunction-associated steatohepatitis-induced hepatocellular carcinoma, altered cholesterol and squalene metabolism can contribute to anti-PD-1 resistance, whereas targeting squalene epoxidase may restore therapeutic efficacy (28). Thus, lipid mediators connect dietary fat composition, host metabolic disease, liver inflammation, and immune checkpoint response.

### 2.3 Cellular targets in the TIME

The third layer of this framework consists of cellular targets within and around the TIME. Nutritional mediators can act on tumor cells, CD8^+^ T cells, Tregs, MDSCs, TAMs, neutrophils, CAFs, adipocytes, hepatocytes, endothelial cells, and microbial communities. The final immune outcome depends on how these compartments interact. CD8^+^ T cells are central targets of nutritional regulation. Their antitumor function requires metabolic flexibility, mitochondrial activity, amino acid availability, and resistance to exhaustion. Nutritional interventions may enhance CD8^+^ T-cell infiltration, cytokine production, cytotoxicity, or memory-like features. FMD can improve antitumor immune responses in low-immunogenic breast cancer models (21). Fructose-mediated adipocyte metabolism can drive antitumor CD8^+^ T-cell responses (32). Galactose-related hepatocyte reprogramming may prevent CD8^+^ T-cell dysfunction and promote antitumor immunity (24).

Myeloid cells are equally important. MDSCs and TAMs frequently suppress T-cell function through arginine depletion, reactive oxygen species production, immunosuppressive cytokines, lipid accumulation, and checkpoint ligand expression. Nutritional and metabolic interventions may reduce myeloid suppression or reprogram myeloid cells toward immunostimulatory states. Sulforaphane can activate CD8^+^ T-cell antitumor responses through IL-12RB2/MMP3/FasL-induced MDSC apoptosis (33). Leucine can promote antigen-presenting neutrophil states and enhance anti-PD-1 efficacy (26). In MASH-associated hepatocellular carcinoma, targeting squalene epoxidase may reduce immunosuppressive myeloid activity and restore anti-PD-1 response (28).

Stromal cells also serve as key targets of nutritional regulation. CAFs can produce CXCL12, remodel extracellular matrix, restrict T-cell infiltration, and create immune-excluded niches. Ketogenesis-mediated suppression of KLF5-dependent CXCL12 production provides an example of how a nutritional metabolic state can reshape stromal immune exclusion (22). Similarly, FMD may influence both tumor and stromal compartments, thereby improving immune responsiveness in otherwise poorly immunogenic tumors (21). Metabolic organs, especially adipose tissue and liver, should also be included in the conceptual framework. Adipocytes can release lipids, adipokines, inflammatory mediators, and metabolites that regulate immune-cell behavior. Fructose-mediated adipocyte metabolism and microbial metabolite-driven adipocyte lipid mobilization illustrate how adipose tissue can act as a systemic regulator of antitumor immunity. Hepatocytes may also shape systemic immune tone and CD8^+^ T-cell exhaustion through diet-derived metabolic programs, as shown in galactose-related immune regulation (24). Therefore, the TIME should not be considered an isolated local compartment; it is connected to whole-body metabolism.

### 2.4 Immune outcomes

The fourth layer of this framework consists of immune outcomes. Nutritional regulation may enhance or suppress antitumor immunity through several mechanisms: increased CD8^+^ T-cell infiltration, improved T-cell effector function, reduced T-cell exhaustion, enhanced antigen presentation, decreased MDSC or TAM-mediated suppression, altered neutrophil function, weakened CAF-mediated immune exclusion, and improved response to PD-1/PD-L1 blockade. Conversely, some dietary exposures may impair immune surveillance or reduce immunotherapy efficacy by disrupting the microbiota, causing nutrient deficiency, or reinforcing immunosuppressive cell states. Positive immune outcomes have been observed in multiple experimental contexts. FMD and short-term starvation-like interventions may enhance antitumor immunity and improve immunotherapy response (21, 34). Ketogenesis may attenuate immunosuppressive chemokine signaling and improve checkpoint blockade response. TMAO, omega-3-related lipid pathways, sulforaphane, leucine, galactose, and microbial FAD-related pathways have all been linked to improved antitumor immune activity or enhanced ICB efficacy in specific tumor models (29).

However, negative or context-dependent outcomes are equally important. Methionine restriction-induced sulfur deficiency may impair antitumor immunity (25). Dietary fiber does not consistently enhance ICB efficacy across diverse murine tumor models, suggesting that generalized recommendations may be biologically oversimplified (35). Sucralose can ablate cancer immunotherapy response through microbiome disruption (17). Together, these findings support a bidirectional framework in which nutritional inputs may either promote or impair antitumor immunity depending on the host–microbiota–tumor context. Nutritional inputs generate metabolic mediators through host and microbial pathways; these mediators act on immune, stromal, tumor, and metabolic-organ compartments; and the resulting cellular changes determine whether the TIME becomes more immunostimulatory or more immunosuppressive. For clinical care, this framework also highlights the importance of nursing assessment and education. Patients should be guided away from unsupervised extreme diets or unverified supplements, while clinicians and nurses should recognize nutrition as a potential modifier of immunotherapy response, toxicity, and survivorship. To provide an integrated overview of the current evidence base, **Table 1** summarizes key experimental and translational studies linking nutritional factors, metabolic mediators, TIME remodeling, and immunotherapy response. These studies collectively support the concept that nutrition regulates cancer immunity through systemic metabolism, microbial ecology, stromal remodeling, and immune-cell functional states. **Figure 1** summarizes the central framework of nutritional regulation of the TIME, in which dietary inputs are converted into metabolic mediators that act on immune, stromal, tumor, and metabolic-organ compartments. The figure highlights that nutritional factors may either enhance antitumor immunity or impair immunotherapy response depending on host metabolism, gut microbiota, and tumor context.

**Table 1:** Core experimental and translational studies linking nutrition, metabolism, TIME remodeling, and immunotherapy response.

Nutritional factor or intervention	Cancer type/model	Main metabolic mediator or pathway	Major immune/stromal target	Main effect on TIME or immunotherapy response	PMID
Fasting-mimicking diet	Patients with cancer; translational immune-metabolic analysis	Glucose, insulin, IGF-1, systemic metabolic remodeling	Circulating immune cells; antitumor immune responses	FMD was feasible and safe and reshaped systemic metabolism and antitumor immunity	34789537
Fasting/FMD	Low-immunogenic breast cancer models	Fasting-like metabolic stress; tumor and stromal remodeling	CD8^+^ T cells; immune-suppressive microenvironment	Fasting rendered poorly immunogenic breast cancer more responsive to immunotherapy	36001966
Ketogenesis/ketogenic intervention	Colorectal cancer models	β-hydroxybutyrate; KLF5-CXCL12 axis	CAFs; stromal chemokine network; T-cell exclusion	Ketogenesis attenuated CXCL12 production and relieved immune suppression	35247887
Diet-driven microbiome ecology	Patients receiving cancer immunotherapy	Dietary pattern; microbial ecology; microbiome function	Gut microbiota; systemic immune tone	Diet-associated microbial ecology was linked to immunotherapy outcomes	36138151
Microbiome-derived TMAO	Pancreatic cancer models	TMAO; microbiota-host metabolic axis	Antigen-presenting and effector immune cells	TMAO promoted immune activation and improved ICB response	36083892
Hydrogen sulfide	Colon cancer models	H_2_S; PPARγ/miR-21/PTEN pathway	Immune-cell infiltration; suppressive immune balance	H_2_S created a more favorable immune microenvironment	36525473
Methionine restriction	Tumor-bearing mouse models	Sulfur deficiency; gut microbiota-dependent metabolism	Antitumor immune responses	Methionine restriction impaired antitumor immunity through sulfur deficiency	37537369
Cyclic ketogenic diet or β-hydroxybutyrate	Prostate cancer ICB-resistant models	β-hydroxybutyrate; HDAC-related epigenetic remodeling	CD8^+^ T cells; macrophages; antigen presentation	Ketogenic intervention enhanced ICB response in resistant prostate cancer models	37609341
Dietary fructose	Tumor models with adipocyte involvement	Adipocyte metabolic remodeling; leptin/mTORC1-related signaling	Adipocytes; CD8^+^ T cells	Fructose-mediated adipocyte metabolism supported antitumor CD8^+^ T-cell responses	37863051
High-glucose drink	Glioblastoma models	Glucose-driven gut microbiota modulation	Gut microbiota; antitumor immune responses	Short-term high-glucose supplementation stimulated antitumor immunity through microbiota remodeling	37804509
Leucine	Multiple tumor models; neutrophil profiling	Leucine availability; antigen-presenting neutrophil program	Neutrophils; CD8^+^ T-cell priming	Leucine promoted antigen-presenting neutrophils and enhanced anti-PD-1 response	38447573
Omega-3 PUFA plus sEH inhibition	Tumor immunotherapy models	Omega-3-derived lipid mediators; soluble epoxide hydrolase	Inflammation-resolution pathways; immune effector cells	Omega-3 PUFA and sEH inhibition enhanced cancer immunotherapy	38330013
Sulforaphane	Tumor-bearing models	IL-12RB2/MMP3/FasL axis	MDSCs; CD8^+^ T cells	Sulforaphane induced MDSC apoptosis and restored CD8^+^ T-cell antitumor activity	38296593
Fasting-mimicking diet	Colorectal cancer models	FMD-induced gut microbiota remodeling	Gut microbiota; intestinal and tumor immunity	FMD remodeled microbiota and suppressed colorectal cancer progression	38918380
Squalene epoxidase targeting	MASH-induced hepatocellular carcinoma	Cholesterol/squalene metabolism; SQLE pathway	MDSCs; CD8^+^ T cells; liver immune microenvironment	Targeting SQLE restored anti-PD-1 efficacy in MASH-HCC	38744443
Dietary fiber	Diverse murine tumor models treated with ICB	Fiber-microbiota interaction	Gut microbiota; systemic antitumor immunity	Dietary fiber did not consistently improve ICB efficacy across models	40540354
Sucralose	Mouse tumor models and immunotherapy-related clinical samples	Microbiome disruption; microbial immune-metabolic pathways	Gut microbiota; ICB-responsive immune states	Sucralose consumption impaired cancer immunotherapy response	40742298
Diet-derived galactose	Tumor models involving liver metabolic regulation	Hepatocyte metabolic reprogramming	Hepatocytes; exhausted CD8^+^ T cells	Galactose enhanced antitumor immunity by limiting CD8^+^ T-cell exhaustion	40781141
Microbial metabolite FAD	Tumor models treated with anti-PD-1	Microbial FAD; adipocyte lipid remodeling	Adipocytes; CD8^+^ T-cell immunity	FAD mobilized adipocyte lipid remodeling and enhanced anti-PD-1 therapy	41570815
Short-term starvation	Hepatocellular carcinoma models treated with anti-PD-L1	Starvation-induced extracellular vesicle signaling	TAMs; myeloid immune remodeling	Short-term starvation boosted anti-PD-L1 therapy through macrophage remodeling	39879585

**Figure 1:**
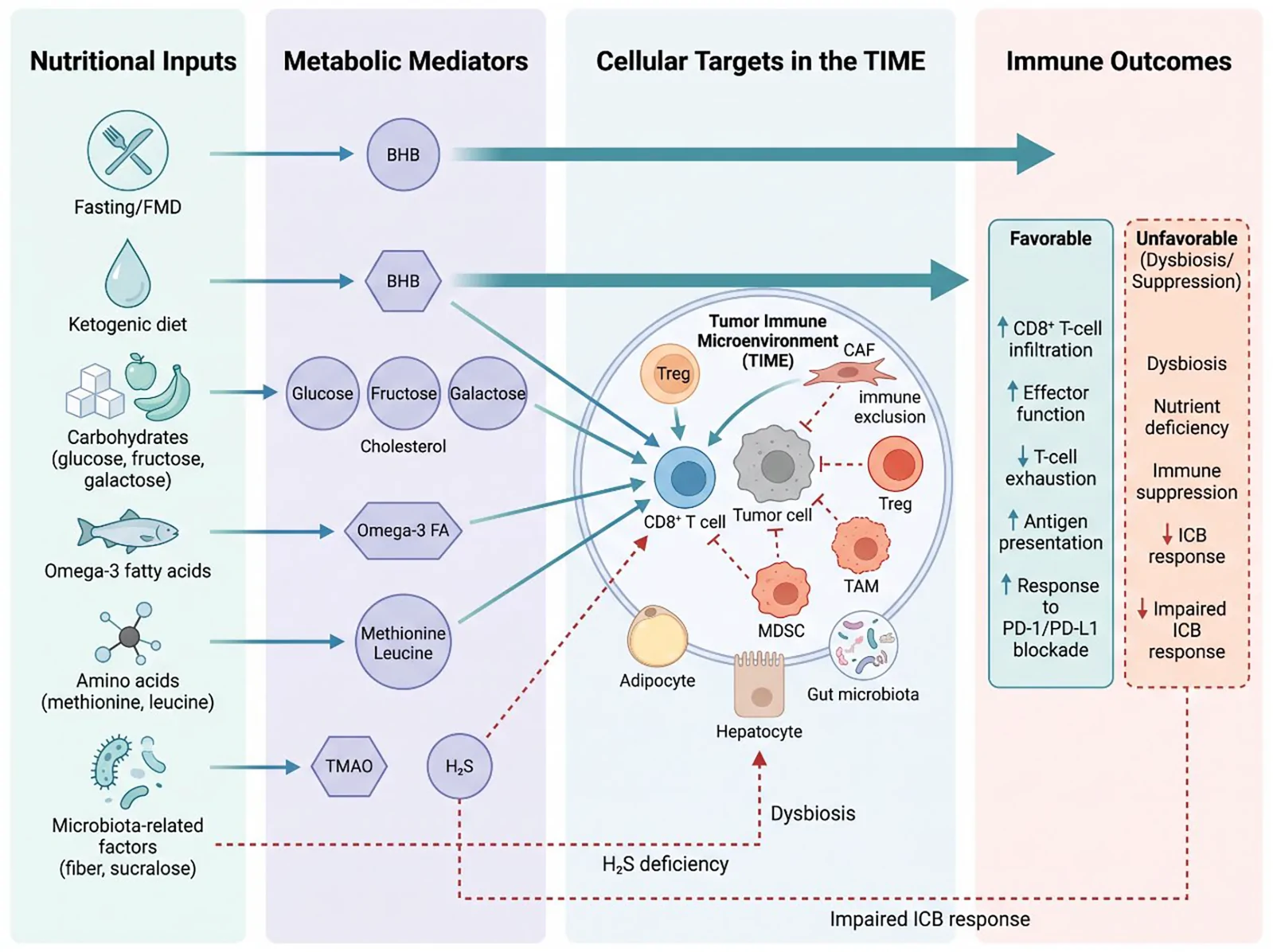
Nutritional regulation of the tumor immune microenvironment. Nutritional inputs, including fasting/FMD, ketogenic diet, carbohydrates, amino acids, lipids, dietary fiber, and artificial sweeteners, are converted into metabolic mediators through host metabolism and gut microbiota. These mediators regulate tumor cells, CD8^+^ T cells, myeloid cells, CAFs, adipocytes, and hepatocytes, thereby reshaping immune suppression, T-cell fitness, stromal exclusion, and response to PD-1/PD-L1 blockade.

### 2.5 Evidence hierarchy and translational interpretation

The evidence summarized in this review should be interpreted according to study type and translational maturity (36). *In vitro* studies are useful for identifying candidate metabolic pathways, immune-cell mechanisms, and nutrient-responsive signaling events, but they cannot capture whole-body metabolism, gut microbiota, tissue-specific nutrient availability, treatment toxicity, or patient behavior. Animal models provide important mechanistic and therapeutic insights, but their dietary composition, microbiome structure, tumor implantation site, immune background, and metabolic physiology may differ substantially from human cancer (37, 38). Observational cohorts can reveal associations between dietary patterns, microbiota, metabolic biomarkers, and immunotherapy outcomes, but they cannot establish causality (39). Early-phase clinical studies may support feasibility, safety, and biological activity, whereas randomized clinical trials and guideline-based recommendations provide stronger evidence for clinical implementation. Therefore, this review distinguishes mechanistic hypotheses from clinically actionable recommendations and avoids direct extrapolation from preclinical findings to routine dietary practice.

## 3 Energy restriction, fasting-mimicking diet, and short-term starvation

Among nutritional interventions, energy restriction, fasting-mimicking diet (FMD), and short-term starvation have received particular attention because they can rapidly alter systemic metabolism and immune-cell behavior (40–44). Unlike conventional nutritional support, which primarily aims to maintain body weight and prevent malnutrition, fasting-like interventions are designed to create a transient metabolic state characterized by reduced glucose availability, decreased insulin and insulin-like growth factor-1 (IGF-1) signaling, enhanced fatty acid oxidation, increased ketone-body production, and altered inflammatory tone. These systemic changes may influence tumor cells, stromal cells, gut microbiota, and immune cells simultaneously. Therefore, fasting-like interventions provide an important model for understanding how nutritional stress can reshape the tumor immune microenvironment (TIME). However, fasting-like interventions should not be interpreted as simple caloric deprivation. Their biological effects depend on timing, duration, nutrient composition, tumor type, host nutritional reserve, treatment context, and immune status. In cancer care, this distinction is essential because patients may confuse structured fasting-mimicking interventions with unsupervised prolonged starvation. From an immunologic perspective, the goal is not to deprive the patient of nutrition, but to induce a controlled and reversible metabolic state that may improve antitumor immunity or treatment sensitivity.

### 3.1 Clinical and translational evidence of FMD

FMD is a periodic, low-calorie, plant-based dietary intervention designed to mimic the metabolic effects of fasting while providing limited nutrients. Compared with complete fasting, FMD is generally more acceptable and potentially safer because it maintains a defined intake of calories, carbohydrates, fats, proteins, and micronutrients. Its major biological rationale is that transient reduction of nutrient-sensing pathways may weaken tumor-supportive metabolic signaling while preserving or enhancing antitumor immune function. Clinical and translational evidence has begun to support this concept. Vernieri and colleagues showed that FMD was feasible and safe in patients with cancer and was associated with systemic metabolic remodeling, including changes in glucose, insulin, IGF-1, and other circulating metabolic parameter (15). Importantly, the study also suggested that FMD could influence antitumor immune responses, indicating that dietary intervention may have immunologic consequences beyond weight control or energy restriction (45–49). This finding is significant because it provides human evidence that structured nutritional modulation can interact with cancer immunity. Mechanistically, FMD may affect the TIME through several interconnected routes. First, reduced glucose and insulin/IGF-1 signaling can create metabolic stress in tumor cells and may limit growth-promoting pathways. Second, fasting-like conditions may alter the availability of amino acids and lipids, thereby changing metabolic competition between tumor cells and immune cells. Third, systemic metabolic changes may improve immune-cell fitness by reducing chronic inflammatory signaling and enhancing stress-resistance programs. Fourth, FMD may affect circulating cytokines and immune-cell composition, thereby altering the balance between effector and suppressive immunity (50–54). These effects may collectively promote a more immunostimulatory microenvironment, although the magnitude and direction of response are likely tumor-type dependent. From a translational perspective, FMD is particularly attractive because it can be scheduled around anticancer therapy cycles. This may allow temporary metabolic modulation without requiring long-term dietary restriction. Nevertheless, the clinical evidence remains early. Larger prospective trials are needed to determine which patients benefit most, which tumor types are most responsive, whether FMD can improve immune checkpoint blockade response, and how safety should be monitored in patients with advanced disease, cachexia, diabetes, or gastrointestinal symptoms.

### 3.2 FMD and immunotherapy sensitization

A major reason for interest in FMD is its potential to sensitize tumors to immunotherapy. Many tumors fail to respond to immune checkpoint blockade because they are immunologically “cold” or immune-excluded, with limited T-cell infiltration, poor antigen presentation, stromal barriers, and dominant immunosuppressive cell populations. FMD may counteract some of these barriers by inducing metabolic and immune remodeling within the tumor microenvironment. Cortellino and colleagues demonstrated that fasting could render immunotherapy effective against low-immunogenic breast cancer (21). This study is important because low-immunogenic tumors are usually poorly responsive to immune checkpoint blockade. The findings suggest that fasting-like metabolic stress can convert an immunologically resistant tumor context into a more therapy-responsive state. FMD may act by altering tumor-cell metabolism, reducing immunosuppressive signals, and enhancing T-cell-mediated antitumor responses. In this sense, FMD functions not merely as a dietary intervention, but as a metabolic adjuvant that may improve the efficacy of immunotherapy.

The mechanisms underlying FMD-mediated immunotherapy sensitization are likely multifactorial. Reduced nutrient availability may increase tumor-cell stress and immunogenicity, making tumor cells more visible to immune attack (55–59). At the same time, changes in systemic metabolism may enhance cytotoxic T-cell function or reduce suppressive myeloid-cell activity. FMD may also affect the stromal compartment. In immune-excluded tumors, CAFs and extracellular matrix remodeling can physically and chemically restrict T-cell access to cancer cells. If fasting-like interventions reduce stromal immunosuppression or modify chemokine gradients, they may facilitate immune-cell infiltration and improve checkpoint blockade response. This concept is highly relevant to the broader field of nutritional immuno-oncology. Immunotherapy resistance is often explained by tumor genetics, antigen presentation defects, checkpoint ligand expression, or immune-cell exhaustion. FMD introduces an additional layer: the host nutritional-metabolic state may determine whether immune cells can function effectively within the tumor. Therefore, nutritional interventions may be most useful when combined with immunotherapy, chemotherapy, or targeted therapy rather than used as standalone anticancer strategies.

### 3.3 FMD-gut microbiota axis

The gut microbiota represents a central interface between nutritional intervention and tumor immunity. Dietary changes can rapidly reshape microbial composition, microbial-derived metabolites, intestinal barrier function, and systemic immune tone. Because the gut microbiota influences response to immune checkpoint blockade, FMD-mediated microbial remodeling may be one mechanism by which nutritional intervention affects the TIME. Luo and colleagues reported that FMD remodeled the gut microbiota and suppressed colorectal cancer progression (60). This study provides important mechanistic support for the FMD-gut microbiota-immune axis. In colorectal cancer, the relationship between diet, microbiota, and local immunity is particularly relevant because the tumor develops in direct contact with intestinal microbial communities. FMD may alter the abundance or function of specific bacterial taxa, change microbial metabolites, and modulate immune-cell activation in the intestine and tumor microenvironment. These changes may contribute to tumor suppression and may potentially interact with anti-PD-1 therapy.

The FMD-gut microbiota axis also helps explain why dietary interventions may produce variable outcomes across patients. Individuals differ in baseline microbiota composition, dietary habits, antibiotic exposure, gastrointestinal function, and inflammatory status (61–65). Therefore, the same FMD protocol may produce distinct microbial and immune consequences in different patients. This variability has major clinical implications. It suggests that future FMD-based strategies may need to incorporate microbiome profiling, dietary history, antibiotic use, and gastrointestinal toxicity monitoring. Importantly, the microbiota-mediated effects of FMD should not be reduced to the idea that “fasting improves the microbiome.” Instead, FMD appears to impose a temporary ecological pressure on the gut microbial community. Depending on the baseline microbial ecosystem, this pressure may enrich beneficial immune-supportive pathways or, in some contexts, worsen dysbiosis. Therefore, clinical translation should be cautious and biomarker-guided.

### 3.4 Short-term starvation and myeloid remodeling

Short-term starvation is another fasting-like intervention that may influence tumor immunity (66–70). Compared with FMD, short-term starvation usually involves more severe and brief nutrient deprivation. Its effects may include acute metabolic stress, altered circulating metabolites, increased autophagy-related responses, and changes in extracellular vesicle signaling. These changes can influence not only tumor cells and lymphocytes, but also myeloid cells, which are major regulators of immunotherapy resistance. Cheng and colleagues showed that short-term starvation could boost anti-PD-L1 therapy in hepatocellular carcinoma through mechanisms involving extracellular vesicles and tumor-associated macrophage remodeling (34). This finding is notable because hepatocellular carcinoma often develops in metabolically and immunologically complex liver environments, where macrophages, Kupffer cells, myeloid-derived suppressor cells, and hepatic metabolic pathways interact closely. Short-term starvation may alter communication between tumor cells and immune cells, thereby reducing myeloid-mediated suppression and improving anti-PD-L1 efficacy.

Myeloid remodeling is a particularly important mechanism because many tumors resist immunotherapy through suppressive macrophages and MDSCs rather than through T-cell-intrinsic defects alone (71–74). TAMs can inhibit T-cell infiltration, express immune checkpoint ligands, secrete immunosuppressive cytokines, promote angiogenesis, and support tumor invasion. If short-term starvation can reprogram TAM function or alter extracellular vesicle-mediated signaling, it may shift the TIME from a suppressive state toward a more permissive antitumor immune state. Nevertheless, starvation-based approaches raise important safety concerns. Patients with advanced cancer may already have anorexia, muscle wasting, liver dysfunction, impaired glycogen reserve, or treatment-related gastrointestinal toxicity. In such patients, even short-term starvation could worsen fatigue, hypoglycemia, dehydration, sarcopenia, or treatment intolerance. Therefore, short-term starvation should be considered an experimental metabolic intervention rather than a general clinical recommendation.

Clinically, fasting-like interventions should be distinguished from unsupervised starvation. Although FMD and short-term starvation may remodel systemic metabolism and antitumor immunity in selected settings, patients with cachexia, sarcopenia, diabetes, liver or renal dysfunction, poor oral intake, or active gastrointestinal toxicity may be vulnerable to harm. Therefore, fasting-related behaviors should be screened before and during immunotherapy and referred for multidisciplinary nutritional evaluation when risk factors are present. **Figure 2** illustrates how controlled fasting-like interventions reshape systemic metabolism and transmit these changes to the TIME through tumor cells, gut microbiota, myeloid cells, stromal barriers, and CD8^+^ T cells. It also highlights the clinical need to distinguish structured FMD or short-term metabolic intervention from unsafe, unsupervised starvation.

**Figure 2:**
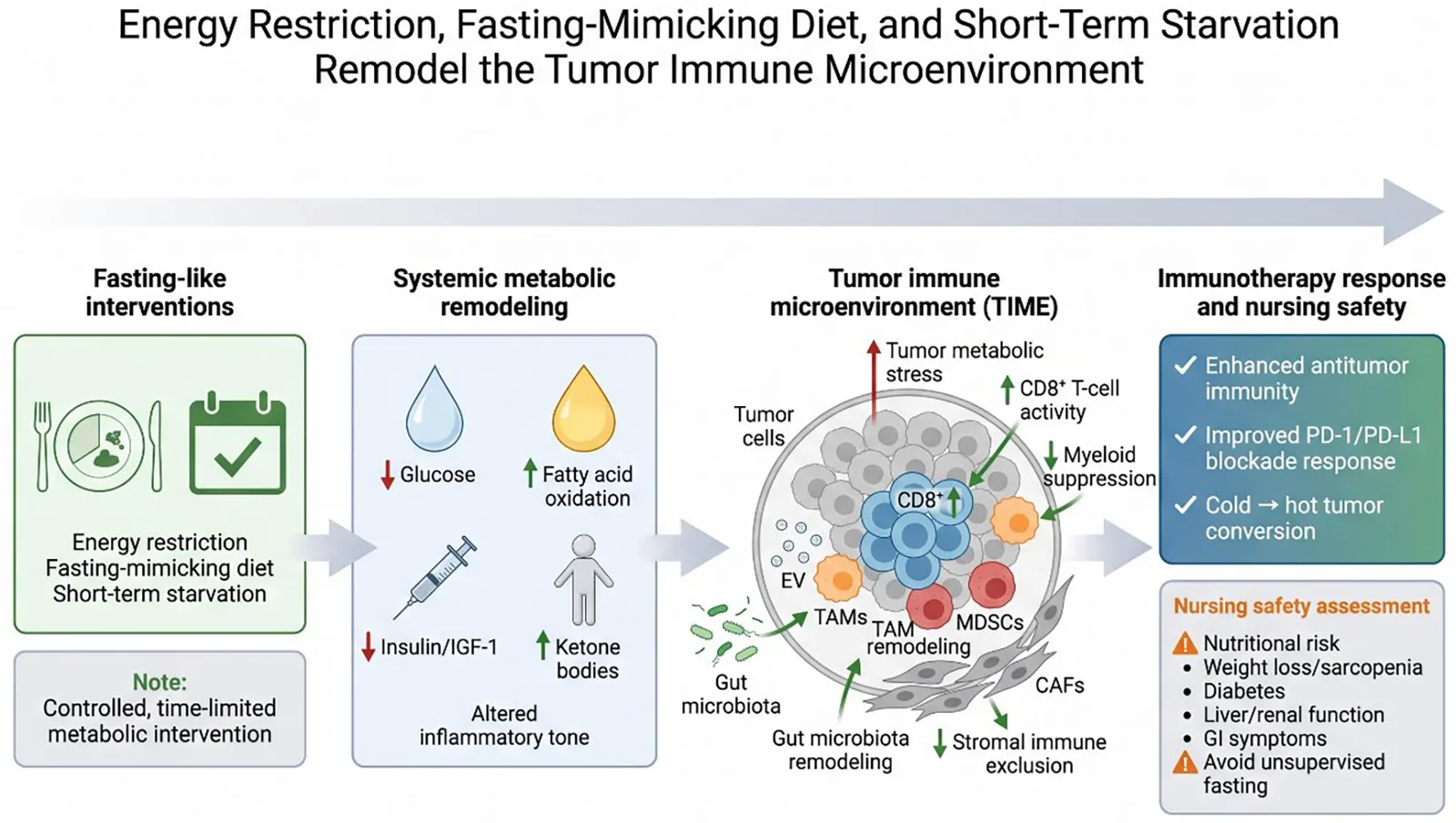
Fasting-like interventions remodel the tumor immune microenvironment. Energy restriction, fasting-mimicking diet, and short-term starvation induce transient systemic metabolic changes, including reduced glucose/insulin/IGF-1 signaling and increased ketone-body production. These changes regulate tumor-cell stress, gut microbiota, TAM/MDSC activity, CAF-mediated stromal barriers, and CD8^+^ T-cell function, thereby enhancing antitumor immunity and potentially improving PD-1/PD-L1 blockade response.

## 4 Ketogenic diet and ketone-body-mediated immune remodeling

Ketogenic diets are high-fat, very-low-carbohydrate dietary patterns designed to shift systemic metabolism from glucose utilization toward fatty acid oxidation and ketone-body production. The major circulating ketone bodies include β-hydroxybutyrate, acetoacetate, and acetone, among which β-hydroxybutyrate has received the greatest attention as a signaling metabolite. In oncology, ketogenic diets were initially discussed mainly in terms of tumor energy metabolism, based on the idea that some tumors depend heavily on glucose metabolism. However, recent studies indicate that ketogenic interventions may also regulate cancer immunity, stromal signaling, and response to immune checkpoint blockade. The immunologic effects of ketogenic diets are complex. On one hand, reduced glucose availability may limit tumor glycolysis and modify metabolic competition within the TIME. On the other hand, ketone bodies may act as signaling molecules that regulate inflammation, epigenetic states, oxidative stress, and immune-cell function. Therefore, ketogenic diets should not be understood only as “glucose restriction.” Rather, they represent a broader metabolic intervention that changes the balance among glucose, lipids, ketone bodies, insulin signaling, and inflammatory mediators.

### 4.1 Ketogenesis and CAF-mediated immune suppression

CAFs are major stromal regulators of immune exclusion and immunotherapy resistance. They can remodel extracellular matrix, secrete immunosuppressive cytokines and chemokines, and restrict cytotoxic T-cell access to tumor nests. Among CAF-derived factors, CXCL12 is especially important because it can contribute to T-cell exclusion and formation of an immune-suppressive stromal niche (75–79). Nutritional modulation of CAF signaling therefore represents an important mechanism by which diet may reshape the TIME. Wei and colleagues showed that ketogenesis attenuates KLF5-dependent production of CXCL12 and helps overcome the immunosuppressive tumor microenvironment in colorectal cancer (22). This study provides a mechanistic link between ketone-body metabolism and stromal immune regulation. Rather than acting only on tumor-cell proliferation, ketogenesis influenced the stromal chemokine network that controls immune-cell localization and function. By suppressing KLF5-dependent CXCL12 production, ketogenesis reduced an immune-excluding signal and promoted a more favorable antitumor immune context (80–84). This finding is important for several reasons. First, it expands the role of ketogenic metabolism from tumor-cell energy restriction to TIME remodeling. Second, it highlights CAFs as metabolically responsive cells that can translate systemic dietary states into local immune consequences. Third, it suggests that ketone-body-mediated signaling may improve immunotherapy response by weakening stromal barriers rather than directly activating T cells alone. In tumors where CAF-rich stroma limits immune-cell infiltration, ketogenic or ketone-related interventions may therefore have particular relevance. Mechanistically, β-hydroxybutyrate may function as more than an alternative fuel. It can influence transcriptional regulation, inflammatory signaling, and cellular stress responses. In the context of colorectal cancer, ketogenesis-mediated modulation of the KLF5-CXCL12 axis suggests that metabolic interventions can rewire chemokine landscapes within the TIME. This provides a rationale for combining ketogenic strategies with immune checkpoint blockade, especially in tumors characterized by immune exclusion.

### 4.2 Ketogenic diet and immune checkpoint resistance

Immune checkpoint resistance remains a major challenge in tumors with low baseline immunogenicity, limited T-cell infiltration, or suppressive myeloid and stromal compartments. Ketogenic diets may influence checkpoint response by altering systemic metabolism, tumor-cell stress, antigen presentation, cytokine signaling, and adaptive immune function. In this setting, ketone bodies may act as metabolic and signaling mediators that improve the ability of immune cells to attack tumors. Murphy and colleagues reported that ketogenic diet-based intervention could overcome prostate cancer resistance to immune checkpoint blockade. In this study, cyclic ketogenic diet or β-hydroxybutyrate supplementation enhanced the efficacy of immune checkpoint therapy. This is particularly relevant because prostate cancer is often considered relatively immunologically cold and poorly responsive to immune checkpoint blockade. The finding suggests that metabolic reprogramming through ketogenic intervention may improve immune responsiveness even in tumors that are not classically sensitive to immunotherapy.

The mechanisms may involve several layers. Ketogenic diet-induced β-hydroxybutyrate may influence T-cell function, tumor-cell signaling, and inflammatory pathways. It may also modulate epigenetic regulation because β-hydroxybutyrate can affect histone modification and transcriptional programs (85–88). Through these effects, ketogenic intervention may enhance adaptive antitumor immunity, reduce immune suppression, or increase tumor vulnerability to immune attack. Importantly, the cyclic nature of ketogenic intervention may be relevant because intermittent metabolic pressure may avoid some of the risks associated with chronic severe dietary restriction. This evidence supports the idea that ketogenic diets should be studied as immunometabolic modulators rather than as universal anticancer diets. Their benefit is likely to depend on tumor type, baseline metabolic phenotype, host body composition, microbiota, immune status, and treatment combination. In some patients, ketogenic diets may induce weight loss, dyslipidemia, constipation, micronutrient imbalance, or reduced dietary adherence. Therefore, clinical translation requires careful patient selection and monitoring. From a clinical perspective, ketogenic interventions should be discussed as investigational immunometabolic modulators rather than universal anticancer diets. Strict carbohydrate restriction may worsen weight loss, constipation, micronutrient imbalance, dyslipidemia, or poor treatment tolerance in vulnerable patients. Therefore, ketogenic diet use, exogenous ketones, medium-chain triglyceride products, and related supplements should be documented and managed with dietitian and oncology-team input. **Figure 3** illustrates how ketogenic diet-induced ketone-body production, especially β-hydroxybutyrate, may reshape the TIME by weakening CAF-mediated immune exclusion and enhancing CD8^+^ T-cell access to tumor cells. It also emphasizes that ketogenic interventions should be considered immunometabolic modulators rather than universal anticancer diets.

**Figure 3:**
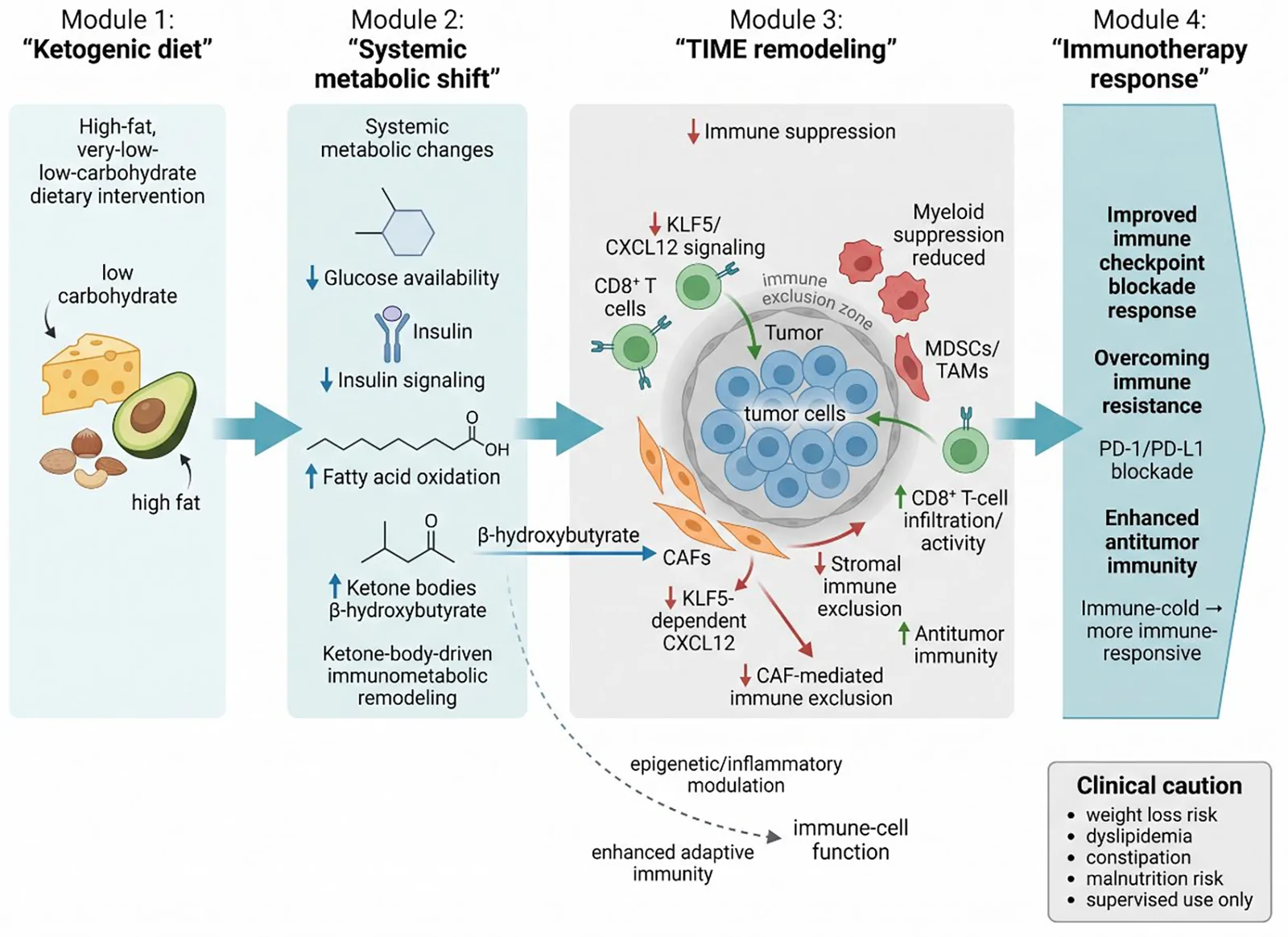
Ketogenic diet-mediated remodeling of the tumor immune microenvironment. Ketogenic diet shifts systemic metabolism from glucose utilization toward fatty acid oxidation and ketone-body production, particularly β-hydroxybutyrate. Ketogenesis may suppress CAF-associated KLF5/CXCL12 signaling, reduce stromal immune exclusion, enhance CD8^+^ T-cell infiltration and activity, and improve PD-1/PD-L1 blockade response, while requiring careful clinical monitoring for nutritional and metabolic risks.

## 5 Carbohydrate-derived signals: glucose, fructose, galactose, and sweeteners

Carbohydrate-derived signals represent one of the most misunderstood areas in cancer nutrition. In public and clinical discussions, carbohydrates are often simplified into the concept that “sugar feeds cancer” and that carbohydrate restriction is therefore universally beneficial. However, recent immunometabolic studies suggest a more complex and context-dependent picture. Different carbohydrate sources, including glucose, fructose, galactose, and non-nutritive sweeteners, can exert distinct effects on tumor immunity depending on dose, duration, tumor type, host metabolic organs, gut microbiota, and immune-cell state (89–93). Therefore, carbohydrate-related nutrition should not be discussed only in terms of tumor growth energetics, but also in terms of immune regulation. Within the tumor immune microenvironment (TIME), carbohydrate availability can influence both cancer cells and immune cells. Tumor cells frequently consume large amounts of glucose through aerobic glycolysis, which may create metabolic competition with effector T cells. At the same time, immune cells require glucose and mitochondrial substrates to sustain proliferation, cytokine production, and cytotoxic activity. In addition, carbohydrate intake can indirectly regulate immunity through adipose tissue, liver metabolism, intestinal microbiota, and systemic inflammatory tone. Thus, the immunologic consequence of carbohydrate exposure cannot be predicted solely by the amount of sugar consumed. Instead, carbohydrate-derived signals should be understood as part of a broader host–microbiota–tumor metabolic network.

### 5.1 Fructose-adipocyte-CD8^+^ T-cell axis

Fructose has traditionally been viewed negatively in metabolic disease because excessive intake is associated with obesity, insulin resistance, fatty liver disease, and chronic inflammation. In cancer biology, fructose is also often discussed as a potential tumor-promoting nutrient. However, recent evidence indicates that fructose may have more complex effects on antitumor immunity, particularly through adipose tissue metabolism. Zhang and colleagues showed that dietary fructose-mediated adipocyte metabolism can drive antitumor CD8^+^ T-cell responses (32). This finding is important because it shifts attention away from a tumor-cell-centered view of fructose metabolism and toward a host-organ-mediated immune mechanism. In this model, adipocytes serve as metabolic intermediaries that translate dietary fructose exposure into immune-regulatory signals. Rather than acting only as passive fat-storage cells, adipocytes can influence T-cell function through adipokines, lipid handling, nutrient release, and inflammatory mediators.

The fructose-adipocyte-CD8^+^ T-cell axis highlights several conceptual points. First, adipose tissue should be considered an active immune-metabolic organ in cancer (94–98). Changes in adipocyte metabolism can affect the availability of lipids, cytokines, and endocrine signals that influence T-cell fitness. Second, the effect of fructose on tumor immunity may depend on host metabolic state. In lean hosts, obese hosts, or patients with metabolic syndrome, adipocyte responses to fructose may differ substantially. Third, this study cautions against oversimplified conclusions that all sugar exposure uniformly suppresses antitumor immunity. A dietary substrate may have harmful systemic metabolic effects in one context but may engage immune-supportive pathways in another experimental setting. Nevertheless, this evidence should not be interpreted as support for high-fructose intake in patients with cancer. The experimental finding is mechanistically valuable, but clinical translation requires caution. Long-term excessive fructose consumption remains associated with metabolic dysfunction, and metabolic dysfunction itself can promote inflammation, fatty liver disease, and treatment complications. Therefore, the key message is not that fructose should be recommended, but that carbohydrate-derived immune regulation is biologically nuanced and mediated through host metabolic organs.

### 5.2 High-glucose drink and glioblastoma immunity

Glucose is the most widely discussed carbohydrate in cancer metabolism. Because many tumors exhibit increased glucose uptake and glycolytic activity, glucose restriction has often been proposed as a potential anticancer strategy (99–103). However, glucose also supports immune-cell activation, and systemic glucose availability may influence the gut microbiota and neuroimmune interactions. This is particularly relevant in glioblastoma, where the brain, gut microbiota, systemic metabolism, and immune system interact in complex ways. Kim and colleagues reported that supplementation with a high-glucose drink stimulated antitumor immune responses to glioblastoma through gut microbiota modulation (23). This study provides a striking example of context-dependent nutritional immunology. Although high sugar intake is generally considered metabolically unfavorable, short-term high-glucose supplementation in this experimental context altered the gut microbiota and promoted antitumor immune responses. The finding challenges a simplistic “glucose always worsens cancer” narrative and suggests that timing, duration, tumor type, and microbial ecology are critical determinants of immune outcome.

The mechanism is particularly interesting because the effect was mediated through the gut microbiota rather than direct glucose action on tumor cells alone. Dietary glucose may change microbial composition or microbial metabolite production, which in turn can regulate systemic immunity and antitumor responses. In glioblastoma, where local immune infiltration is limited and systemic immune modulation may be important, microbiota-mediated effects may have substantial relevance (104–108). This also supports the broader concept that nutritional interventions can influence distant tumors through gut-derived immune signals. However, this finding should be interpreted carefully in clinical contexts. It does not justify recommending high-glucose beverages to patients with glioblastoma or other cancers. Patients with cancer may have diabetes, steroid-induced hyperglycemia, insulin resistance, cachexia, or infection risk, and uncontrolled glucose intake may worsen metabolic complications. The value of this study lies in its mechanistic message: carbohydrate effects on cancer immunity are not determined only by tumor glucose consumption, but also by host–microbiota–immune interactions.

### 5.3 Galactose-hepatocyte-CD8^+^ T-cell exhaustion axis

Galactose is less commonly discussed than glucose or fructose in cancer nutrition, but emerging data suggest that it may have distinctive immunometabolic effects (109–114). Unlike glucose, galactose enters specific hepatic and systemic metabolic pathways and may influence liver-derived signals that regulate immune-cell function. Because the liver is a central metabolic and immune-tolerogenic organ, diet-derived hepatic reprogramming may have important consequences for systemic antitumor immunity. Recent evidence indicates that diet-derived galactose can reprogram hepatocytes and enhance antitumor CD8^+^ T-cell immunity by limiting T-cell exhaustion (24). This study provides another example of how a dietary carbohydrate can regulate tumor immunity indirectly through a host metabolic organ. In this model, hepatocytes act as a metabolic sensor and immune-regulatory hub. By responding to galactose-derived metabolic cues, hepatocytes may alter systemic signals that preserve CD8^+^ T-cell function and prevent exhaustion-associated immune failure. This mechanism is highly relevant to immunotherapy because CD8^+^ T-cell exhaustion is a major determinant of poor response to immune checkpoint blockade. Exhausted T cells exhibit reduced cytokine production, impaired cytotoxicity, altered transcriptional programs, and sustained expression of inhibitory receptors. If dietary or metabolic interventions can reduce exhaustion or improve T-cell functional persistence, they may increase sensitivity to PD-1/PD-L1 blockade. The galactose-hepatocyte-CD8^+^ T-cell axis therefore expands the concept of nutritional immunotherapy beyond direct immune-cell nutrient uptake. The broader implication is that metabolic organs can determine the immune consequences of dietary inputs. The liver integrates carbohydrate, lipid, amino acid, bile acid, and inflammatory signals. In patients with fatty liver disease, viral hepatitis, alcohol-related liver disease, or hepatic metastasis, the same dietary carbohydrate may produce different immune outcomes. Therefore, future studies should investigate whether liver metabolic status modifies the immunologic effects of galactose and other carbohydrate-derived signals.

### 5.4 Artificial sweeteners and immunotherapy failure

Artificial sweeteners are highly relevant to oncology nursing because many patients reduce sugar intake by switching to “sugar-free” beverages, low-calorie products, or diabetic-friendly foods (115–118). These products are often perceived as metabolically neutral because they contain little or no caloric sugar. However, non-nutritive sweeteners may influence host metabolism and gut microbiota, which can have downstream effects on antitumor immunity and immunotherapy response. Morder and colleagues showed that sucralose consumption can ablate cancer immunotherapy response through microbiome disruption (17). This study is particularly important because it directly connects a common dietary additive with impaired response to cancer immunotherapy. The mechanism involves disruption of microbial ecology and immune-metabolic pathways rather than simply caloric intake. Thus, “sugar-free” does not necessarily mean immunologically neutral. This finding has several implications. First, dietary assessment in patients receiving immunotherapy should not focus only on sugar intake, caloric intake, or macronutrient distribution. It should also include artificial sweeteners, diet beverages, low-calorie snacks, sugar substitutes, and commercial nutrition products. Second, microbiome disruption may be a major hidden pathway through which dietary additives influence treatment efficacy. Third, patient education should address the misconception that replacing sugar with artificial sweeteners is always beneficial. The sucralose study also underscores the need for caution in dietary recommendations. Patients with cancer often receive simplified advice to “avoid sugar,” which may lead them to consume large amounts of artificial sweeteners. If certain sweeteners disrupt the microbiota and reduce immunotherapy efficacy, such advice could unintentionally produce harm. Therefore, dietary counseling should be individualized and evidence-informed rather than based on broad anti-sugar messages. These findings support a balanced clinical message: carbohydrate-derived signals should not be reduced to the slogan that “sugar feeds cancer.” Glucose, fructose, galactose, and artificial sweeteners may influence antitumor immunity through distinct host-organ and microbiota-dependent pathways. During immunotherapy, nurses should document major carbohydrate restriction, high-sugar intake, fruit avoidance, diet beverages, and artificial sweetener use, while avoiding both high-sugar recommendations and fear-based anti-sugar counseling. **Figure 4** illustrates that carbohydrate-derived signals should not be viewed simply as tumor fuel, but as context-dependent immunometabolic regulators acting through adipocytes, hepatocytes, and gut microbiota. The figure highlights both immune-supportive pathways, such as enhanced CD8⁺ T-cell function, and unfavorable pathways, such as sucralose-induced dysbiosis and reduced ICB response.

**Figure 4:**
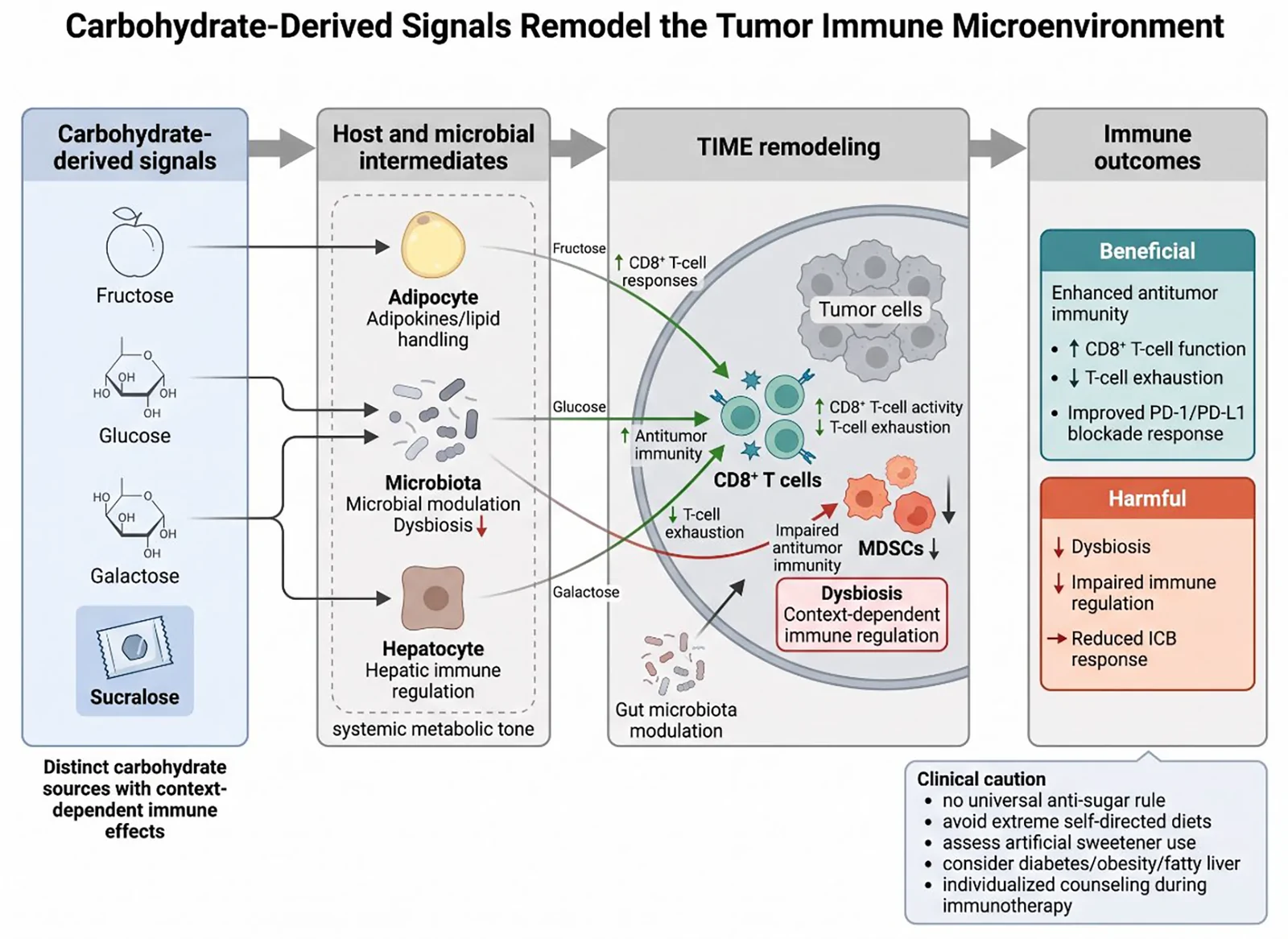
Carbohydrate-derived signals regulate tumor immunity through host–microbiota interactions. Glucose, fructose, galactose, and artificial sweeteners exert distinct immune effects through gut microbiota, adipocytes, and hepatocytes. Fructose may enhance adipocyte-mediated CD8^+^ T-cell responses, glucose may modulate microbiota-dependent immunity, and galactose may reduce CD8^+^ T-cell exhaustion, whereas sucralose-induced dysbiosis may impair antitumor immunity and reduce ICB response.

## 6 Amino acid and sulfur metabolism in antitumor immunity

Amino acids are essential regulators of cancer immunity. They provide substrates for protein synthesis, nucleotide synthesis, redox balance, epigenetic modification, one-carbon metabolism, and immune-cell proliferation. Effector T cells, antigen-presenting cells, myeloid cells, and tumor cells all require amino acids, but they use them in different ways (119–122). As a result, amino acid availability can influence metabolic competition within the TIME and determine whether immune responses are activated or suppressed. Amino acid metabolism is especially relevant to immunotherapy because immune-cell activation requires rapid metabolic remodeling. T cells need amino acids to expand, produce cytokines, and maintain cytotoxic function. Myeloid cells can suppress antitumor immunity by depleting amino acids such as arginine or by generating immunosuppressive metabolites through pathways such as indoleamine 2,3-dioxygenase-related tryptophan metabolism. Sulfur-containing amino acids, including methionine and cysteine, are also linked to glutathione synthesis, methylation reactions, hydrogen sulfide production, and redox homeostasis. Therefore, amino acid-targeted nutritional strategies may have both beneficial and harmful effects depending on the immune context.

### 6.1 Methionine restriction: beneficial or harmful?

Methionine restriction has attracted considerable interest in cancer metabolism because many tumors are sensitive to changes in methionine availability. Methionine is involved in one-carbon metabolism, S-adenosylmethionine production, methylation reactions, nucleotide synthesis, and redox balance (123–127). Restricting methionine may therefore disrupt tumor growth and alter epigenetic or metabolic programs. However, recent evidence indicates that methionine restriction may also impair antitumor immunity under certain conditions. Ji and colleagues showed that methionine restriction-induced sulfur deficiency can impair antitumor immunity through gut microbiota-dependent mechanisms (25). This study is highly important because it challenges the assumption that nutrient restriction is always beneficial in cancer. Although restricting a tumor-supportive nutrient may appear attractive, the same restriction may deprive immune cells or microbiota-derived immune pathways of necessary metabolic substrates. In this case, sulfur deficiency altered host–microbiota interactions and weakened antitumor immune responses (128–133). This finding has broad conceptual significance. First, anticancer nutritional restriction must be evaluated from both tumor-cell and immune-cell perspectives. A nutrient that supports tumor growth may also be required for immune defense. Second, the gut microbiota can determine the immunologic consequences of amino acid restriction. Methionine restriction does not act only on tumor or host cells; it can reshape microbial metabolism, sulfur availability, and systemic immune tone. Third, amino acid restriction may have different effects depending on baseline diet, microbiota composition, tumor type, and treatment regimen.

For clinical translation, methionine restriction should therefore be viewed with caution. Patients may independently attempt low-protein or amino acid-restricted diets after reading about cancer metabolism. However, such restrictions may worsen malnutrition, sarcopenia, hypoalbuminemia, fatigue, and immune dysfunction, especially in patients with advanced disease. The key message is that nutritional restriction is not automatically equivalent to immune enhancement. A strategy that suppresses tumor metabolism may simultaneously weaken host immunity if not properly designed and monitored.

### 6.2 Hydrogen sulfide as an immune-regulatory metabolite

Hydrogen sulfide (H_2_S) is traditionally known as a toxic gas, but it is now recognized as an endogenous gasotransmitter involved in vascular regulation, inflammation, mitochondrial function, redox balance, and cellular signaling. In cancer, H_2_S biology is complex and context dependent. It may support tumor metabolism in some settings, but it can also modulate immune responses and reshape the TIME. Yue and colleagues reported that H_2_S creates a favorable immune microenvironment through the PPARγ/miR-21/PTEN pathway in colon cancer (31). This study suggests that sulfur-related metabolites can act as immune-regulatory signals rather than merely metabolic byproducts. Through modulation of the PPARγ/miR-21/PTEN axis, H_2_S may influence immune-cell infiltration, immune suppression, and antitumor immune activity. This provides an important mechanistic link between sulfur metabolism and TIME remodeling.

The relationship between H_2_S and antitumor immunity may involve several mechanisms. H_2_S can regulate mitochondrial function and oxidative stress, both of which are critical for immune-cell survival and activation (134–138). It may also influence macrophage polarization, T-cell activity, cytokine production, and inflammatory signaling. By affecting PTEN-related pathways, H_2_S may modulate cellular metabolism and immune-regulatory programs within tumor and stromal compartments (139, 140). These effects indicate that sulfur metabolism should be incorporated into nutritional immunology frameworks. However, the H_2_S pathway also illustrates the importance of dose and context. Sulfur-containing nutrients, microbial sulfur metabolism, endogenous H_2_S-producing enzymes, and tumor-specific metabolic states may all determine whether H_2_S has immune-supportive or tumor-promoting effects. Therefore, H_2_S should not be framed simply as beneficial or harmful. Instead, it should be discussed as a context-dependent metabolic mediator that connects diet, microbiota, redox biology, and immune regulation.

### 6.3 Leucine and antigen-presenting neutrophils

Leucine is a branched-chain amino acid that regulates protein synthesis, mTOR signaling, immune-cell activation, and metabolic fitness. In cancer nutrition, leucine is often discussed in the context of muscle preservation, sarcopenia prevention, and protein supplementation (141–145). However, recent evidence indicates that leucine may also influence antitumor immunity by reshaping myeloid-cell states. Wu and colleagues showed that leucine can promote antitumor antigen-presenting neutrophil activity and enhance anti-PD-1 therapy (26). This finding is important because neutrophils have traditionally been viewed mainly as inflammatory or tumor-promoting cells in cancer. However, neutrophils are heterogeneous, and specific subsets may acquire antigen-presenting capacity and support antitumor immunity. Leucine availability may help drive or maintain this immunostimulatory neutrophil state (146–149). This mechanism expands the role of nutrition in myeloid-cell reprogramming. While many studies focus on T cells, the efficacy of immunotherapy also depends on antigen presentation, innate immune activation, and the balance between suppressive and stimulatory myeloid populations. If leucine can enhance antigen-presenting neutrophils, then amino acid availability may directly influence the quality of immune priming and checkpoint blockade response. This provides a link between nutritional protein metabolism and innate-adaptive immune cooperation.

The leucine-neutrophil axis also has potential clinical relevance for patients with cancer-related malnutrition or sarcopenia. Protein intake and amino acid supplementation are commonly used to preserve muscle mass, but their immune consequences are less well understood. Leucine may therefore represent a point of convergence between nutritional support and immunotherapy optimization. However, it remains uncertain whether leucine supplementation in patients can reproduce the immune effects observed experimentally. Clinical studies are needed to define dose, timing, safety, tumor specificity, and interaction with immunotherapy. Clinically, amino acid-targeted strategies should be interpreted with caution. Although methionine restriction and leucine availability provide mechanistic insights into tumor–immune metabolism, unsupervised protein or amino acid restriction may worsen malnutrition, sarcopenia, hypoalbuminemia, and immune dysfunction. Protein intake, amino acid supplements, and restrictive diets should therefore be assessed as part of nutritional risk monitoring during immunotherapy. **Figure 5** illustrates how amino acid and sulfur-related metabolic pathways reshape antitumor immunity through gut microbiota, redox signaling, myeloid-cell remodeling, and CD8^+^ T-cell regulation. It emphasizes that amino acid restriction is not uniformly beneficial and should be balanced against the need to preserve immune fitness and nutritional status.

**Figure 5:**
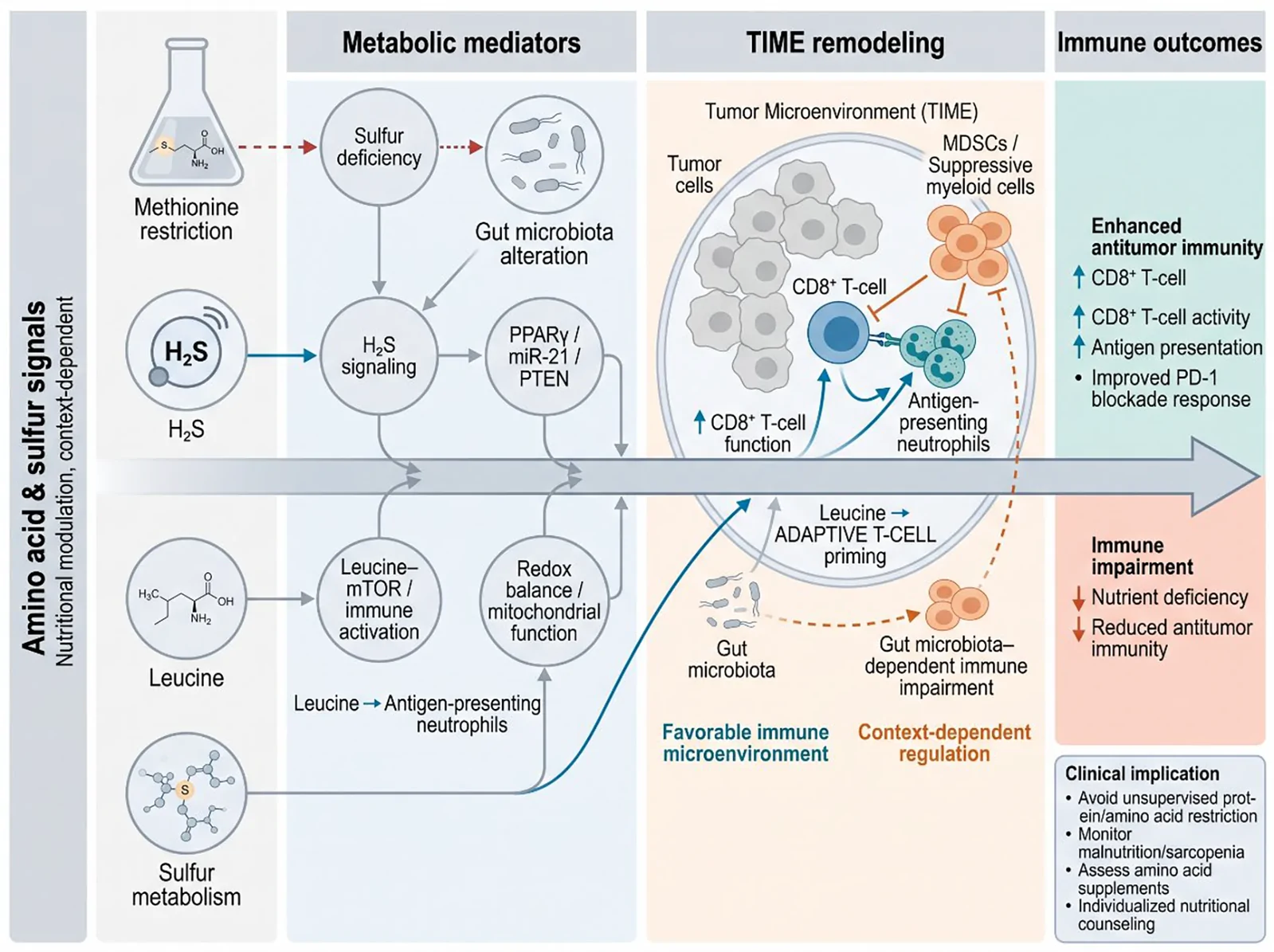
Amino acid and sulfur metabolism regulate antitumor immunity. Methionine restriction, sulfur metabolism, H_2_S signaling, and leucine availability exert context-dependent effects on the TIME. Methionine restriction-induced sulfur deficiency may impair antitumor immunity through gut microbiota-dependent mechanisms, whereas H_2_S signaling and leucine-driven antigen-presenting neutrophils may enhance immune activation, CD8^+^ T-cell function, and anti-PD-1 response.

## 7 Lipid metabolism, adipose tissue, and inflammation resolution

Lipid metabolism is a central component of nutritional regulation of the tumor immune microenvironment (TIME). Lipids are not only structural components of cellular membranes or long-term energy stores; they also function as signaling molecules, inflammatory mediators, substrates for oxidative metabolism, and regulators of immune-cell differentiation. Within tumors, altered lipid uptake, fatty acid oxidation, cholesterol metabolism, lipid peroxidation, and eicosanoid signaling can influence tumor-cell survival, myeloid-cell suppression, T-cell dysfunction, and response to immune checkpoint blockade. The relationship between lipid metabolism and antitumor immunity is highly context dependent. Certain lipid-derived mediators can promote chronic inflammation, myeloid-cell recruitment, T-cell exhaustion, and immune escape. In contrast, other lipid pathways, especially those involved in inflammation resolution, may enhance antitumor immunity and improve immunotherapy response. Adipose tissue also plays an important role in this process. Rather than serving as a passive energy reservoir, adipose tissue functions as an endocrine and immune-metabolic organ that releases adipokines, fatty acids, cytokines, extracellular vesicles, and microbiota-linked metabolites. These signals may reshape systemic immunity and influence local immune states within the TIME.

### 7.1 Omega-3 fatty acids and soluble epoxide hydrolase

Omega-3 polyunsaturated fatty acids (PUFAs) have long been considered anti-inflammatory nutrients. Traditionally, their potential benefits in cancer care have been discussed in relation to inflammation control, body weight maintenance, cachexia, and cardiovascular protection. However, recent evidence suggests that omega-3 fatty acids may also influence cancer immunotherapy through specific lipid mediator pathways. This expands the role of omega-3 PUFAs from general nutritional support to active immunometabolic modulation. Kelly and colleagues showed that omega-3 PUFA supplementation combined with inhibition of soluble epoxide hydrolase (sEH) enhanced cancer immunotherapy (27). This study is important because it identifies a mechanistic lipid pathway that can regulate the antitumor effects of immune checkpoint blockade. sEH metabolizes epoxy fatty acids into less active diols, thereby limiting the activity of lipid mediators involved in inflammation resolution and immune regulation. By inhibiting sEH, omega-3-derived lipid mediators may be preserved or enhanced, promoting a more favorable immune environment for immunotherapy (150–154). This mechanism provides several important insights. First, lipid nutrition should not be viewed only in terms of total fat intake. The type of fatty acid, its downstream enzymatic metabolism, and the balance between pro-inflammatory and pro-resolving lipid mediators are critical. Second, inflammation resolution is not simply immune suppression. In the tumor context, resolving chronic inflammatory signals may reduce myeloid-cell-driven immune suppression and improve effector immune-cell function. Third, lipid mediator pathways may be pharmacologically targetable, suggesting that dietary lipid modulation and metabolic enzyme inhibition could be combined to improve immunotherapy response.

The omega-3/sEH axis also illustrates the potential for nutritional-pharmacologic combinations. Omega-3 fatty acids provide the substrate pool, whereas sEH inhibition modifies downstream lipid mediator availability. Such strategies may be more precise than broad dietary advice because they target defined metabolic pathways. However, clinical translation requires careful evaluation of dose, timing, safety, tumor type, baseline diet, anticoagulant use, and interaction with systemic therapy. In addition, omega-3 supplementation should not be assumed to universally improve immunotherapy outcomes without biomarker-guided validation.

### 7.2 Cholesterol/squalene metabolism in MASH-HCC

Metabolic dysfunction-associated steatohepatitis (MASH) has become an increasingly important background disease in hepatocellular carcinoma (HCC) (155–158). MASH-associated HCC develops within a chronically inflamed, lipid-rich, fibrotic, and metabolically stressed liver microenvironment. This setting differs substantially from viral hepatitis-associated HCC and may influence immune surveillance and response to immune checkpoint blockade. In MASH-HCC, dysregulated lipid metabolism, hepatocyte injury, oxidative stress, myeloid-cell recruitment, and T-cell dysfunction may collectively generate an immunotherapy-resistant environment. Wen and colleagues demonstrated that targeting squalene epoxidase restored anti-PD-1 efficacy in MASH-induced HCC (28). Squalene epoxidase is a key enzyme in cholesterol biosynthesis, and its dysregulation can influence sterol metabolism, lipid accumulation, and immune-cell behavior. The study suggests that cholesterol/squalene metabolism is not merely a hepatocyte metabolic abnormality, but a driver of immune resistance in MASH-HCC (159–163). By targeting this pathway, the investigators were able to reverse immunosuppressive features and improve anti-PD-1 response. This finding is highly relevant because it links host metabolic disease with immunotherapy resistance. Patients with obesity, fatty liver disease, diabetes, dyslipidemia, or MASH may have a different immune-metabolic baseline than patients with non-metabolic tumor etiologies. In MASH-HCC, the liver microenvironment may contain lipid-laden macrophages, dysfunctional T cells, inflammatory hepatocytes, activated stellate cells, and altered bile acid or cholesterol metabolism. These factors can suppress effective antitumor immunity even when checkpoint pathways are pharmacologically blocked.

The cholesterol/squalene pathway also highlights the need to consider metabolic comorbidities in immuno-oncology. Immune checkpoint blockade is often discussed in terms of tumor PD-L1 expression, tumor mutational burden, T-cell infiltration, or molecular subtype. However, host metabolic inflammation may be equally important in certain cancers. In MASH-HCC, targeting lipid metabolism may represent a strategy to recondition the liver immune microenvironment and restore immunotherapy sensitivity. This concept may also extend to other obesity- or lipid-associated cancers, although tumor-specific validation is required.

### 7.3 Adipocyte lipid remodeling and microbial metabolites

Adipose tissue is increasingly recognized as an active regulator of cancer immunity. Adipocytes can influence tumor progression through lipid transfer, adipokine secretion, inflammatory cytokine release, extracellular vesicle communication, and metabolic crosstalk with immune cells (164–168). In obesity and cancer-associated metabolic remodeling, adipocytes may become hypertrophic, inflamed, insulin resistant, or lipolytic, producing signals that affect myeloid cells, T cells, and tumor cells. Therefore, adipose tissue should be considered part of the systemic immune-metabolic network that shapes the TIME. Tong and colleagues reported that the microbial metabolite FAD mobilizes adipocyte lipid remodeling and enhances anti-PD-1 therapy (30). This study is important because it connects gut microbial metabolism, adipose tissue lipid dynamics, and antitumor immunity. Rather than acting directly within the tumor alone, a microbiota-derived metabolite influenced adipocyte lipid handling, which then contributed to improved immune checkpoint blockade response. This provides a clear example of the gut microbiota-adipose tissue-TIME axis. The finding also expands the definition of dietary regulation. Nutritional inputs can alter gut microbial composition and microbial metabolite production; these microbial metabolites can act on distant metabolic organs such as adipose tissue; adipose-derived lipid signals can then regulate immune-cell function and immunotherapy response. This multilayered pathway explains why nutritional interventions may have systemic effects that are not predictable from nutrient composition alone. A dietary factor may influence tumor immunity indirectly by changing microbial metabolites and adipocyte behavior.

Adipocyte lipid remodeling may affect antitumor immunity through several mechanisms. Mobilized fatty acids may serve as metabolic substrates for immune cells or tumor cells, depending on context. Adipokines may regulate T-cell activation, myeloid-cell polarization, and inflammatory tone. Lipid-derived mediators may influence oxidative stress, antigen presentation, or checkpoint responsiveness. Importantly, adipose tissue responses may differ between lean, obese, cachectic, and sarcopenic patients. Thus, body composition and adipose tissue quality may be more informative than body weight alone. These lipid-related findings indicate that host metabolic disease and body composition may modify nutritional effects on antitumor immunity. In clinical care, obesity, sarcopenic obesity, dyslipidemia, fatty liver disease, MASH, omega-3 supplementation, fish oil use, and high-fat ketogenic products should be documented because they may influence immunotherapy tolerance, liver-related adverse-event interpretation, and nutritional risk. **Figure 6** illustrates how lipid-derived mediators, host metabolic disease, and adipose tissue remodeling shape the TIME through inflammation resolution, myeloid-cell regulation, and CD8^+^ T-cell activity. It highlights the dual role of lipid metabolism in either promoting immune resistance or supporting immunotherapy response depending on metabolic context.

**Figure 6:**
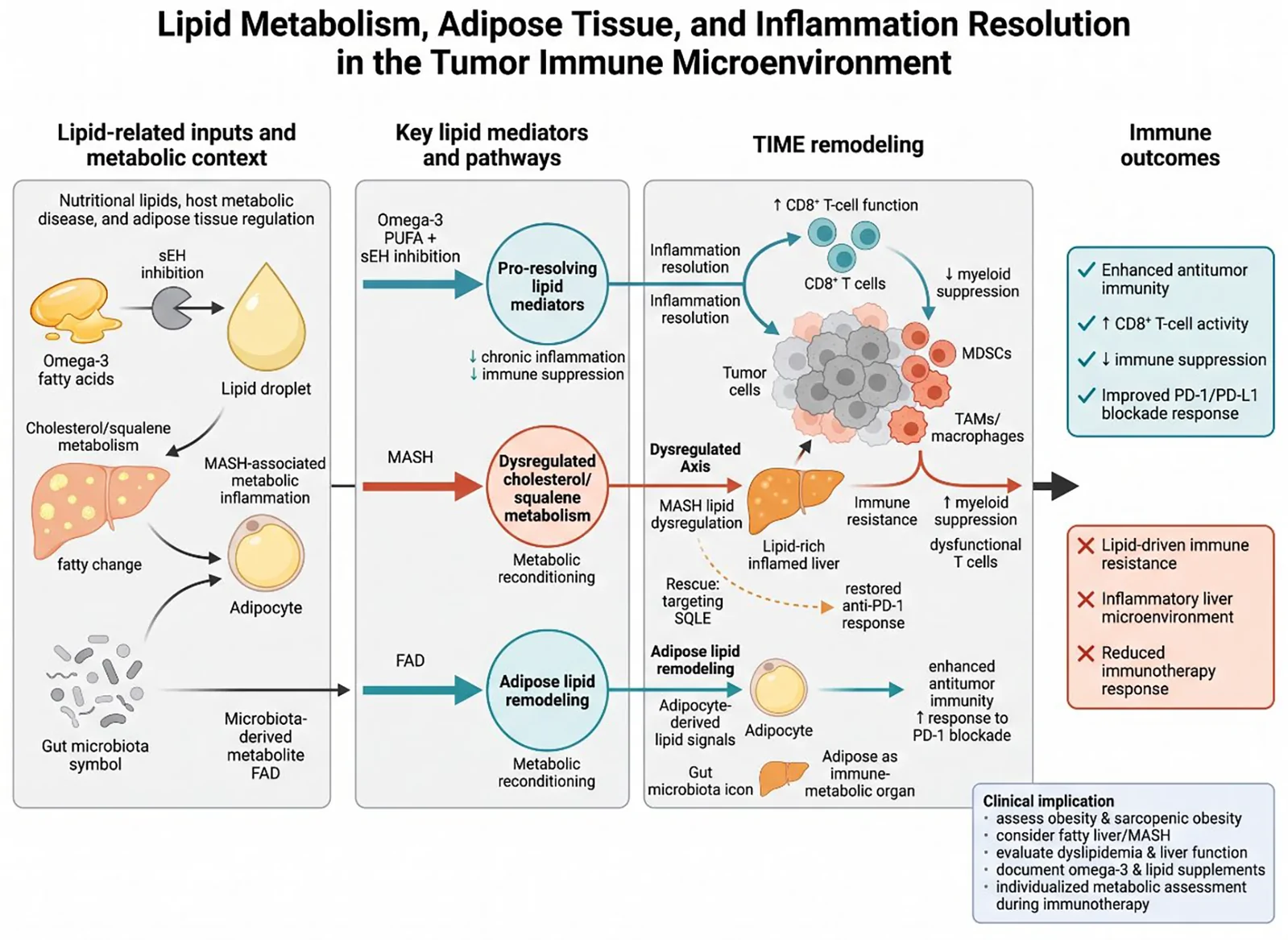
Lipid metabolism and adipose tissue remodeling regulate antitumor immunity. Omega-3 fatty acids and sEH inhibition may promote pro-resolving lipid mediators, reduce myeloid suppression, and enhance CD8^+^ T-cell activity. In contrast, MASH-associated cholesterol/squalene dysregulation can drive immune resistance, whereas microbiota-derived FAD may remodel adipocyte lipid signaling and improve anti-PD-1 response.

## 8 Gut microbiota as the central interface between nutrition and immunotherapy

The gut microbiota is one of the most important biological interfaces connecting nutrition with antitumor immunity (169–172). Dietary inputs can rapidly alter microbial composition, microbial gene expression, metabolite production, intestinal barrier integrity, and mucosal immune tone. These microbial changes can then influence systemic immunity, antigen presentation, T-cell priming, myeloid-cell activity, inflammatory cytokines, and response to immune checkpoint blockade. Therefore, the gut microbiota provides a mechanistic bridge between external nutritional exposure and internal immune regulation. The microbiota is especially relevant to immunotherapy because response to PD-1/PD-L1 and CTLA-4 blockade depends not only on tumor-intrinsic factors, but also on host immune readiness. Specific microbial communities or microbial metabolites may promote dendritic-cell activation, enhance effector T-cell responses, reduce suppressive myeloid activity, and improve ICB efficacy. Conversely, dysbiosis may impair immune priming, increase inflammation, damage intestinal barrier function, or promote treatment resistance. Nutrition is one of the most practical and modifiable factors shaping this microbial-immune axis.

### 8.1 Diet-driven microbial ecology and ICI outcomes

Dietary patterns influence the microbiota at multiple levels, including microbial diversity, abundance of specific taxa, short-chain fatty acid production, bile acid metabolism, amino acid metabolism, and mucosal immune regulation. Because different regions and populations have distinct dietary habits, the relationship between microbiota and immunotherapy response may vary across clinical cohorts. Therefore, understanding diet-driven microbial ecology is essential for interpreting ICB outcomes. Simpson and colleagues showed that diet-driven microbial ecology underpins associations between cancer immunotherapy outcomes and the gut microbiome (16). This study emphasizes that the microbiome should not be analyzed in isolation from diet. Dietary intake shapes microbial communities, and these communities in turn influence immune competence and treatment response (173–178). The findings support a more integrated model in which diet, microbiota, and immunotherapy are biologically connected rather than independent variables. This concept has several implications. First, microbiome biomarkers of ICB response may be difficult to generalize across populations without dietary context. A microbial signature associated with response in one cohort may not have the same meaning in another cohort with different dietary habits, geography, antibiotic exposure, or lifestyle factors. Second, dietary interventions aimed at improving immunotherapy outcomes should be designed with microbiome function in mind, not only nutrient composition. Third, patient-reported dietary patterns may become important covariates in immunotherapy studies and clinical practice.

For nursing and clinical care, this evidence suggests that diet history should be incorporated into immunotherapy assessment. Information about fiber intake, fermented foods, processed foods, artificial sweeteners, supplements, probiotics, antibiotics, and major recent dietary changes may help contextualize gastrointestinal symptoms and treatment response. Although routine microbiome-guided dietary prescriptions are not yet standard, diet-microbiota awareness is increasingly relevant to patient education and supportive care.

### 8.2 Microbiome-derived TMAO in pancreatic cancer

Microbiome-derived metabolites can have highly disease-specific effects. Trimethylamine N-oxide (TMAO) is commonly discussed as a metabolite associated with cardiovascular and metabolic risk. It is generated through microbial metabolism of dietary precursors such as choline, phosphatidylcholine, and carnitine, followed by hepatic conversion. However, its role in cancer immunity may differ from its role in cardiovascular disease. Mirji and colleagues showed that the microbiome-derived metabolite TMAO drives immune activation and boosts responses to immune checkpoint blockade in pancreatic cancer (29). This finding is striking because pancreatic cancer is typically characterized by dense stroma, poor T-cell infiltration, immunosuppressive myeloid cells, and limited responsiveness to immunotherapy. The study suggests that a microbial metabolite often considered metabolically unfavorable in other contexts may enhance antitumor immunity in pancreatic cancer. This example illustrates the principle of disease-context dependence. A metabolite cannot be classified as universally beneficial or harmful without considering the tissue, tumor type, immune context, and therapeutic setting. In pancreatic cancer, TMAO may promote immune activation and improve responsiveness to ICB, whereas in cardiovascular disease it may be interpreted differently. This duality is important for nutritional immunology because many diet-derived metabolites have pleiotropic effects across organ systems.

The TMAO study also reinforces the concept that microbial metabolites can act systemically. Dietary precursors are processed by gut microbes, converted by host metabolic organs, and then influence immune responses at distant tumor sites. Therefore, nutritional regulation of immunotherapy response may involve multi-organ communication among the gut, liver, immune system, and tumor microenvironment. Future studies are needed to determine whether TMAO-related pathways can be safely manipulated in patients, especially those with cardiovascular comorbidities.

### 8.3 Dietary fiber: positive expectation and negative evidence

Dietary fiber is often considered beneficial because it supports microbial diversity, short-chain fatty acid production, intestinal barrier function, and metabolic health. In the context of immunotherapy, fiber has been proposed as a potential dietary strategy to improve gut microbiota composition and enhance ICB response. However, the evidence is not uniformly positive, and recent experimental data highlight the need for caution. Roichman and colleagues reported a lack of consistent effect of dietary fiber on immune checkpoint blockade efficacy across diverse murine tumor models (35). This study is valuable because it provides negative or context-dependent evidence in a field where positive microbiome-diet associations are often emphasized. The findings suggest that dietary fiber does not automatically improve ICB response in all tumor models. Its effects may depend on fiber type, microbial baseline, tumor model, immune context, and treatment regimen. This evidence has important implications for clinical interpretation. Fiber may still be beneficial for general health, bowel function, metabolic regulation, and microbiota support, but it should not be presented as a universal immunotherapy-enhancing intervention. Different fibers produce different microbial metabolites. Soluble and insoluble fibers, resistant starches, inulin-type fructans, and other fermentable substrates may have distinct effects. In addition, patients vary in tolerance, especially during cancer therapy. Some patients may experience bloating, diarrhea, abdominal pain, or worsening gastrointestinal symptoms with high-fiber intake. The negative fiber evidence also strengthens the overall argument of this review: nutritional interventions must be evaluated within specific biological contexts. A dietary factor that appears beneficial in one model may be neutral or even harmful in another. Therefore, recommendations should avoid overgeneralization. Future studies should identify which patients, tumor types, microbiota profiles, and fiber types are most likely to benefit.

### 8.4 Microbiota-mediated dietary risk: sucralose and methionine restriction

The gut microbiota can mediate beneficial effects of nutrition, but it can also mediate dietary risks. This point is especially important because patients often assume that dietary changes or “healthy” substitutions are safe. Two recent examples—sucralose consumption and methionine restriction—demonstrate how diet-microbiota interactions can impair antitumor immunity or immunotherapy response. Sucralose consumption has been shown to ablate cancer immunotherapy response through microbiome disruption (17). This finding is clinically relevant because sucralose is widely used in sugar-free beverages, low-calorie foods, and diabetic products. Patients attempting to reduce sugar intake may inadvertently increase artificial sweetener consumption. If sucralose disrupts microbial ecology and weakens immunotherapy response, then common dietary substitutions may have unintended immune consequences. This does not mean all artificial sweeteners have identical effects, but it does show that non-nutritive dietary additives should not be assumed to be biologically inert.

Methionine restriction provides a second example of microbiota-mediated dietary risk. Although methionine restriction has been explored as a tumor-targeting metabolic strategy, methionine restriction-induced sulfur deficiency can impair antitumor immunity through gut microbiota-dependent mechanisms (25). This finding challenges the idea that nutrient restriction is always beneficial. By altering sulfur availability and microbial metabolism, methionine restriction may weaken host immune defense even if it places metabolic stress on tumor cells. Together, these studies show that the microbiota is not simply a mediator of nutritional benefit. It is a dynamic ecological system that can convert dietary exposures into either immune-supportive or immune-disruptive signals. Therefore, nutritional counseling during immunotherapy should include attention to artificial sweeteners, restrictive diets, supplement use, antibiotic exposure, gastrointestinal symptoms, and major dietary changes. The goal is not to prohibit all dietary modification, but to prevent unsupervised practices that may disrupt microbial and immune homeostasis.

Overall, the gut microbiota provides a central explanatory framework for why nutritional effects on immunotherapy are variable. Diet modifies microbial ecology; microbial ecology modifies metabolite production and immune tone; and immune tone determines whether checkpoint blockade can generate durable antitumor responses. This framework also creates opportunities for future precision nutrition strategies, including microbiome-informed dietary assessment, metabolite-guided intervention, and nursing-led monitoring of diet-related microbial risk during immunotherapy.

## 9 Nutritional regulation of immune and stromal cell states

After discussing individual nutritional factors and metabolic pathways, it is important to integrate these mechanisms at the cellular level. Nutritional regulation of the tumor immune microenvironment (TIME) does not occur through a single nutrient–cell interaction (179–182). Instead, dietary inputs and metabolic mediators reshape the functional states of multiple cell populations, including CD8^+^ T cells, myeloid cells, neutrophils, cancer-associated fibroblasts (CAFs), adipocytes, hepatocytes, and tumor cells. These cell types form an interconnected immune-metabolic ecosystem in which nutrient availability, metabolite signaling, stromal architecture, and immune-cell fitness collectively determine antitumor immunity. This cell-state perspective is essential because the same nutritional intervention may have different effects depending on which cell population is dominant in a specific tumor. For example, in a T-cell-inflamed tumor, nutritional modulation may mainly enhance effector T-cell activity. In a myeloid-rich tumor, its major effect may be to reduce suppressive macrophages or myeloid-derived suppressor cells (MDSCs). In an immune-excluded tumor, the most important effect may occur through CAFs and stromal barriers. Therefore, nutritional immunology should be interpreted through the lens of cellular ecology rather than nutrient exposure alone.

### 9.1 CD8^+^ T cells

CD8^+^ T cells are central mediators of antitumor immunity and major effectors of immune checkpoint blockade. Their ability to infiltrate tumors, recognize antigen, proliferate, produce cytokines, release cytotoxic molecules, and resist exhaustion depends heavily on metabolic fitness. Activated CD8^+^ T cells require glucose, amino acids, mitochondrial function, lipid metabolic balance, and redox stability. Within the TIME, however, these cells often face nutrient deprivation, hypoxia, lactate accumulation, oxidative stress, and inhibitory cytokines. Nutritional regulation may therefore influence CD8^+^ T-cell immunity by altering both systemic metabolic conditions and local tumor metabolic constraints. Fasting-like interventions provide one example. In low-immunogenic breast cancer models, fasting or fasting-mimicking diet enhanced the effectiveness of immunotherapy, suggesting that metabolic stress can convert an immune-resistant tumor into a more immune-responsive state (21). This effect may involve improved T-cell infiltration, enhanced effector function, reduced immunosuppressive signaling, and increased tumor vulnerability to immune attack. Importantly, this mechanism suggests that nutritional intervention may act as an immune-sensitizing strategy rather than simply a tumor-starving approach.

Carbohydrate-derived signals can also regulate CD8^+^ T-cell states through host metabolic organs. Dietary fructose-mediated adipocyte metabolism has been shown to drive antitumor CD8^+^ T-cell responses (32). This finding indicates that adipocytes can act as intermediaries between dietary substrates and T-cell immunity. Rather than directly feeding tumor cells or immune cells alone, fructose may alter adipose tissue metabolism, which then modifies systemic signals that support CD8^+^ T-cell function. Similarly, diet-derived galactose can reprogram hepatocytes and prevent CD8^+^ T-cell exhaustion, thereby enhancing antitumor immunity (24). These studies highlight the importance of distant metabolic organs in sustaining T-cell fitness. Plant-derived bioactive compounds may further influence CD8^+^ T-cell activity indirectly by suppressing immunosuppressive myeloid populations. Sulforaphane has been reported to activate CD8^+^ T-cell antitumor responses through IL-12RB2/MMP3/FasL-induced MDSC apoptosis (33). In this context, CD8^+^ T-cell recovery is not caused by direct nutrient stimulation alone, but by removal of myeloid-mediated suppression. This illustrates an important principle: nutritional factors may enhance T-cell immunity by remodeling the surrounding immune ecosystem.

Together, these studies suggest that CD8^+^ T cells are metabolically sensitive but not nutritionally autonomous. Their function is shaped by tumor-cell metabolism, stromal exclusion, myeloid suppression, adipocyte signaling, hepatocyte-derived cues, and microbial metabolites. Therefore, nutritional strategies designed to enhance CD8^+^ T-cell immunity should aim to improve the entire metabolic environment in which T cells operate.

### 9.2 Myeloid cells: MDSCs, TAMs, and neutrophils

Myeloid cells are major regulators of nutritional effects on the TIME. MDSCs, tumor-associated macrophages (TAMs), and neutrophils can either suppress or support antitumor immunity depending on their differentiation state, metabolic program, and local cytokine environment. In many tumors, myeloid cells suppress T-cell function through arginine depletion, reactive oxygen species, nitric oxide, lipid accumulation, immunosuppressive cytokines, checkpoint ligand expression, and extracellular matrix remodeling. Nutritional interventions may therefore improve immunotherapy response by reprogramming myeloid-cell states. MDSCs are particularly important because they create a metabolically hostile environment for T cells. Sulforaphane-induced MDSC apoptosis provides an example of how a diet-derived plant compound can relieve myeloid-mediated immune suppression and restore CD8^+^ T-cell activity (33). This mechanism links nutritional bioactive compounds with antitumor immunity through indirect immune remodeling. Rather than simply stimulating effector cells, sulforaphane reduces an inhibitory cell population that prevents T-cell function.

Macrophages are another major target of nutritional-metabolic regulation. In hepatocellular carcinoma, short-term starvation was shown to boost anti-PD-L1 therapy through mechanisms involving extracellular vesicles and tumor-associated macrophage remodeling (34). This finding suggests that acute nutritional stress can alter tumor–macrophage communication and shift macrophage behavior toward a state more compatible with immunotherapy response. Since TAMs are frequently associated with immune exclusion, angiogenesis, tumor invasion, and checkpoint resistance, their remodeling may be a key mechanism through which fasting-like interventions enhance therapy. Metabolic disease-associated myeloid dysfunction is also important. In metabolic dysfunction-associated steatohepatitis-induced hepatocellular carcinoma, targeting squalene epoxidase restored anti-PD-1 efficacy (28). This study links cholesterol/squalene metabolism with myeloid-cell-mediated immune resistance. It suggests that lipid metabolic abnormalities in the liver microenvironment can generate suppressive immune states that limit checkpoint blockade efficacy. Correcting these metabolic pathways may therefore recondition the myeloid compartment and improve immunotherapy response.

Neutrophils are increasingly recognized as heterogeneous immune cells with both tumor-promoting and antitumor functions. Leucine has been shown to promote antitumor antigen-presenting neutrophil activity and enhance anti-PD-1 therapy (26). This finding is important because it expands the role of amino acid metabolism beyond T-cell activation and muscle maintenance. Leucine availability may support a neutrophil state capable of antigen presentation and immune activation, thereby linking nutritional protein metabolism with innate-adaptive immune cooperation. Collectively, these studies indicate that nutritional modulation of myeloid cells is a major mechanism of immune remodeling. In some cases, nutrition reduces suppressive myeloid populations; in others, it reprograms macrophages or neutrophils toward immunostimulatory phenotypes. This helps explain why nutritional interventions may be particularly effective when combined with immune checkpoint blockade, which depends on relief of both T-cell-intrinsic and myeloid-mediated suppression.

### 9.3 CAFs and stromal barriers

CAFs are essential stromal regulators of immune exclusion. They remodel extracellular matrix, produce chemokines, secrete growth factors, alter tissue stiffness, and form physical and biochemical barriers that restrict immune-cell access to tumor cells. In immune-excluded tumors, CD8^+^ T cells may be present at the tumor margin but unable to penetrate tumor nests. Therefore, nutritional regulation of CAF activity and stromal architecture may be an important mechanism for improving immunotherapy response. Fasting-like interventions may influence stromal suppression. In low-immunogenic breast cancer, fasting rendered immunotherapy more effective, in part by altering tumor and microenvironmental conditions that normally limit immune activation (21). Although the immune effects of fasting are often discussed in relation to T cells, stromal remodeling may also be important. Reduced growth factor signaling, altered cytokine production, and metabolic stress may change how CAFs interact with immune cells and tumor cells.

Ketogenesis provides a more direct example of stromal immune regulation. Ketogenesis attenuated KLF5-dependent CXCL12 production and helped overcome the immunosuppressive tumor microenvironment in colorectal cancer (22). CXCL12 is a key chemokine associated with immune exclusion and stromal barrier formation. By suppressing the KLF5-CXCL12 axis, ketogenesis weakened a CAF-related immunosuppressive signal and promoted a more immune-permissive environment. This finding shows that ketone-body-related metabolism can reshape the chemokine landscape rather than simply altering tumor-cell energy metabolism. This CAF-centered mechanism is important for several reasons. First, it expands nutritional immunology beyond immune cells. Nutritional interventions can influence stromal cells, and stromal changes can determine whether immune cells can physically and functionally access tumor cells. Second, it suggests that metabolic interventions may be especially relevant for desmoplastic or immune-excluded tumors. Third, it supports combination strategies in which nutritional or metabolic interventions are used to weaken stromal barriers before or during immunotherapy.

However, CAFs are heterogeneous. Some CAF subsets promote immune exclusion, whereas others may restrain tumor growth or support tissue structure. Therefore, nutritional strategies targeting CAFs must be interpreted carefully. Future studies should define which CAF subsets respond to fasting, ketogenesis, lipid metabolites, or microbial metabolites, and whether these changes improve T-cell infiltration without promoting tumor invasion or fibrosis.

### 9.4 Metabolic organs as immune regulators

A major conceptual advance in nutritional immuno-oncology is the recognition that metabolic organs regulate tumor immunity from a distance. Adipose tissue, liver, skeletal muscle, and gut can shape systemic immune tone through metabolites, hormones, cytokines, bile acids, lipid mediators, and microbial products. Among these organs, adipose tissue and liver have received particular attention because they directly respond to dietary inputs and strongly influence inflammation, nutrient availability, and immune-cell metabolism. Adipose tissue is not a passive reservoir of excess calories. It is an immune-metabolic organ that releases adipokines, fatty acids, inflammatory cytokines, and extracellular vesicles. Dietary fructose-mediated adipocyte metabolism has been shown to drive antitumor CD8^+^ T-cell responses, indicating that adipocytes can translate carbohydrate exposure into immune-regulatory signals (32). Similarly, the microbial metabolite FAD can mobilize adipocyte lipid remodeling and enhance anti-PD-1 therapy (30). These findings support the existence of a gut microbiota-adipose tissue-immune axis that can influence checkpoint blockade response.

The liver is another critical metabolic-immune organ. It integrates carbohydrate, lipid, amino acid, bile acid, and microbial-derived signals. It also maintains immune tolerance under physiological conditions, which may influence systemic antitumor immunity. Diet-derived galactose has been reported to reprogram hepatocytes and prevent CD8^+^ T-cell exhaustion (24). This finding suggests that hepatocyte metabolic states can influence T-cell function at the systemic level. It also raises the possibility that liver disease, fatty liver, hepatitis, or hepatic metastasis may modify the immune effects of dietary interventions. These studies collectively suggest that the TIME should not be viewed as a purely local compartment. It is connected to whole-body metabolism. Nutritional interventions may act through remote organs before their effects are observed in tumors. This concept is especially important for patients with obesity, diabetes, fatty liver disease, cachexia, or sarcopenic obesity. In such patients, the same dietary intervention may produce different immune outcomes because the metabolic organs that interpret nutritional inputs are already altered. Understanding metabolic organs as immune regulators also creates opportunities for precision nutrition. Future studies may stratify patients by body composition, liver fat, insulin resistance, microbiome profile, circulating metabolites, or adipokine patterns. Such stratification may help identify which patients are likely to benefit from fasting-like interventions, ketogenic strategies, lipid modulation, amino acid supplementation, or microbiome-directed diets.

## 10 Nutritional modulation of immunotherapy response

Immune checkpoint blockade has transformed cancer therapy, but many patients fail to respond or eventually develop resistance. Traditional explanations for poor response include low tumor mutational burden, impaired antigen presentation, lack of T-cell infiltration, immune checkpoint redundancy, suppressive myeloid cells, stromal exclusion, and T-cell exhaustion. Nutritional regulation introduces an additional layer of complexity: host metabolic state, dietary exposure, gut microbiota, and nutrient-derived metabolites may influence whether immunotherapy can generate an effective antitumor response. Nutritional modulation of immunotherapy should be understood as a bidirectional process. Some nutritional strategies may improve immune checkpoint blockade by enhancing T-cell fitness, reducing myeloid suppression, weakening stromal barriers, improving antigen presentation, or activating beneficial microbial metabolites. Conversely, other dietary exposures may impair immunotherapy by disrupting the microbiota, inducing nutrient deficiency, worsening metabolic inflammation, or reinforcing immune suppression. Therefore, the goal is not to identify a universal anticancer diet, but to define context-specific immunometabolic strategies that can be safely integrated with cancer treatment.

### 10.1 Nutritional strategies that may enhance ICB

Several nutritional or diet-related interventions have been reported to enhance immune checkpoint blockade in experimental or translational settings. Fasting-like interventions are among the most studied. FMD was shown to be feasible and safe in patients with cancer and to reshape systemic metabolism and antitumor immunity (15). In low-immunogenic breast cancer, fasting rendered immunotherapy effective, suggesting that metabolic intervention can sensitize tumors that are otherwise poorly responsive to immune checkpoint blockade (21). Short-term starvation has also been reported to boost anti-PD-L1 therapy in hepatocellular carcinoma through extracellular vesicle-related mechanisms and macrophage remodeling (34). Together, these studies suggest that fasting-like metabolic stress may enhance immunotherapy through systemic metabolic remodeling, tumor stress responses, and immune-cell reprogramming. Ketogenic interventions represent another potential strategy. Ketogenesis attenuated KLF5-dependent CXCL12 production and helped overcome an immunosuppressive microenvironment in colorectal cancer (22). In prostate cancer models, ketogenic diet or β-hydroxybutyrate supplementation enhanced immune checkpoint blockade response. These findings suggest that ketone-body-mediated metabolism may improve immunotherapy by reducing stromal immune exclusion, altering inflammatory pathways, and supporting adaptive immunity.

Lipid-related strategies may also enhance ICB. Omega-3 PUFA supplementation combined with soluble epoxide hydrolase inhibition improved cancer immunotherapy by modulating lipid mediator pathways involved in inflammation resolution (27). This suggests that lipid metabolism can be therapeutically shaped to create a more favorable immune environment. Similarly, targeting squalene epoxidase restored anti-PD-1 efficacy in MASH-induced HCC, indicating that correction of cholesterol/squalene metabolic abnormalities may overcome metabolic disease-associated immune resistance (28). Microbiota-derived and diet-associated metabolites provide additional opportunities. TMAO enhanced immune activation and boosted immune checkpoint blockade response in pancreatic cancer (29). Leucine promoted antigen-presenting neutrophil states and enhanced anti-PD-1 therapy (26). Sulforaphane activated CD8^+^ T-cell antitumor responses by inducing MDSC apoptosis (33). Diet-derived galactose improved CD8^+^ T-cell immunity by limiting exhaustion through hepatocyte reprogramming (24). The microbial metabolite FAD mobilized adipocyte lipid remodeling and enhanced anti-PD-1 therapy (30). These studies show that nutritional enhancement of ICB can occur through diverse mechanisms, including T-cell preservation, myeloid reprogramming, stromal remodeling, adipocyte lipid mobilization, hepatocyte signaling, and microbial metabolite activity. Despite these promising findings, it is important to note that most of these strategies are not yet ready for routine clinical use. Their effects are highly dependent on tumor type, timing, dose, host metabolism, microbiota composition, and treatment context. Nevertheless, they provide a mechanistic foundation for future precision nutritional immunotherapy.

### 10.2 Nutritional factors that may impair ICB

Not all nutritional interventions improve immunotherapy response. Some may impair antitumor immunity or reduce ICB efficacy. These negative or context-dependent findings are essential because they prevent the field from overpromising dietary strategies and help define safety boundaries for clinical practice. Methionine restriction is a clear example. Although methionine restriction has been explored as an anticancer metabolic strategy, methionine restriction-induced sulfur deficiency impaired antitumor immunity through gut microbiota-dependent mechanisms (25). This finding demonstrates that nutrient restriction can weaken host immune defense even when it appears theoretically attractive from a tumor metabolism perspective. It also shows that the gut microbiota can transform dietary restriction into an immunologically harmful state.

Artificial sweeteners provide another clinically relevant risk. Sucralose consumption ablated cancer immunotherapy response through microbiome disruption (17). This is particularly important because many patients use sugar-free beverages or low-calorie sweeteners during cancer treatment, especially when attempting to reduce sugar intake. The finding indicates that non-nutritive sweeteners should not automatically be considered metabolically or immunologically neutral. Dietary additives may reshape microbial ecology and thereby influence treatment response. Dietary fiber also illustrates the limits of generalized nutritional recommendations. Although fiber is commonly associated with gut health and microbial diversity, dietary fiber did not consistently enhance immune checkpoint blockade efficacy across diverse murine tumor models (35). This does not mean fiber is unimportant for general health or bowel function, but it cautions against presenting fiber as a universal immunotherapy-enhancing intervention. Fiber effects may depend on fiber type, baseline microbiota, tumor model, treatment regimen, and host immune state. These negative and context-dependent studies are scientifically valuable. They show that nutritional immunotherapy is not simply a matter of adding “beneficial” nutrients or removing “harmful” nutrients. Instead, nutritional exposures can produce complex immune consequences. A dietary factor may support immunity in one setting but impair it in another. Therefore, clinical guidance must be based on evidence, patient context, and careful monitoring rather than broad dietary rules. Because nutritional interventions may generate favorable, unfavorable, or context-dependent immune effects, **Table 2** compares dietary factors according to their reported impact on antitumor immunity and immune checkpoint blockade response. This comparison highlights the need to avoid universal dietary prescriptions and instead interpret nutritional strategies within specific tumor, host metabolic, and microbiome contexts.

**Table 2:** Nutritional factors with potentially favorable, unfavorable, or context-dependent effects on antitumor immunity and ICB response.

Category	Nutritional factor or intervention	Proposed mechanism	Potential immune or therapeutic consequence	Key caution	PMID
Potentially favorable	Fasting-mimicking diet	Reduces glucose/insulin/IGF-1 signaling and reshapes systemic immune metabolism	May enhance antitumor immune responses and treatment sensitivity	Should not be confused with unsupervised starvation	34789537
Potentially favorable	Fasting/FMD with immunotherapy	Induces tumor metabolic stress and improves T-cell-associated immunity	May sensitize low-immunogenic tumors to immunotherapy	Safety depends on nutritional reserve and treatment context	36001966
Potentially favorable	Ketogenesis	β-hydroxybutyrate suppresses KLF5-dependent CXCL12 production	May weaken CAF-mediated immune exclusion	Evidence remains tumor-type specific	35247887
Potentially favorable	Cyclic ketogenic diet or β-hydroxybutyrate	Ketone-body-related epigenetic and immune remodeling	May overcome ICB resistance in prostate cancer models	Strict ketogenic diets may worsen weight loss or metabolic complications	37609341
Potentially favorable	TMAO	Microbiome-derived metabolite activates antitumor immunity	May boost ICB response in pancreatic cancer	TMAO has different implications in cardiovascular/metabolic disease	36083892
Potentially favorable	Omega-3 PUFA plus sEH inhibition	Preserves pro-resolving lipid mediators	May enhance immunotherapy through inflammation-resolution pathways	Supplement dose, bleeding risk, and drug interactions require monitoring	38330013
Potentially favorable	Sulforaphane	IL-12RB2/MMP3/FasL-mediated MDSC apoptosis	Restores CD8^+^ T-cell antitumor function	Commercial supplements should not replace evidence-based therapy	38296593
Potentially favorable	Leucine	Supports antigen-presenting neutrophil states	Enhances anti-PD-1 response in experimental models	Clinical dose and patient selection remain undefined	38447573
Potentially favorable	Galactose	Reprograms hepatocyte-derived immune signaling	Limits CD8^+^ T-cell exhaustion and enhances antitumor immunity	Not a rationale for high-sugar diets	40781141
Potentially favorable	Microbial FAD	Mobilizes adipocyte lipid remodeling	Enhances anti-PD-1 response through gut-adipose-immune crosstalk	Requires validation in humans	41570815
Potentially favorable	Short-term starvation	Alters extracellular vesicle signaling and TAM states	Boosts anti-PD-L1 response in HCC models	Unsafe in cachexia, malnutrition, diabetes, or frail patients	39879585
Context-dependent	High-glucose drink	Modulates gut microbiota in GBM models	May stimulate antitumor immunity in specific experimental settings	Should not be translated into clinical high-sugar recommendations	37804509
Context-dependent	Dietary fructose	Reprograms adipocyte metabolism and supports CD8^+^ T-cell responses	May enhance antitumor immunity in selected models	Long-term fructose excess may worsen metabolic disease	37863051
Context-dependent	Hydrogen sulfide	Regulates PPARγ/miR-21/PTEN signaling	May create a favorable immune microenvironment	Dose, source, and tumor context are critical	36525473
Context-dependent	Dietary fiber	Alters microbiota and microbial metabolites	May support gut health, but ICB benefit is inconsistent	Not a universal ICB-enhancing intervention	40540354
Potentially unfavorable	Methionine restriction	Causes sulfur deficiency through microbiota-dependent mechanisms	Impairs antitumor immunity	Nutrient restriction can harm immune fitness	37537369
Potentially unfavorable	Sucralose	Disrupts gut microbiota and immune-metabolic signaling	Reduces cancer immunotherapy response	Artificial sweetener use should be actively assessed	40742298
Context-dependent	Cholesterol/squalene metabolism	SQLE-driven lipid metabolic remodeling in MASH-HCC	Targeting SQLE may restore anti-PD-1 response	Host metabolic disease modifies immunotherapy response	38744443
Context-dependent	Diet-driven microbial ecology	Diet shapes microbial communities and immune tone	May influence ICB outcomes across populations	Geographic, dietary, and antibiotic differences matter	36138151
Context-dependent	FMD-gut microbiota axis	FMD remodels gut microbial ecology	May suppress CRC progression and support antitumor immunity	Microbiome baseline may determine response	38918380

### 10.3 Why evidence is not yet ready for universal dietary prescriptions

Despite rapid progress, nutritional immuno-oncology remains insufficiently mature for universal dietary prescriptions during immunotherapy. The major limitation is not lack of biological plausibility, but the gap between controlled mechanistic models and heterogeneous real-world patients. First, many studies are based on animal models, which do not fully reproduce human dietary habits, microbiota diversity, tumor heterogeneity, metabolic comorbidities, or treatment histories. Second, many interventions are short-term, controlled, and mechanistically designed, whereas real-world patient diets are complex, variable, and influenced by culture, symptoms, socioeconomic factors, and personal beliefs. Third, tumor type matters. A dietary strategy that improves immunotherapy response in pancreatic cancer, colorectal cancer, prostate cancer, hepatocellular carcinoma, or glioblastoma may not have the same effect in melanoma, lung cancer, breast cancer, ovarian cancer, or renal cancer (183–186). Tumors differ in metabolic dependencies, stromal density, immune infiltration, microbiota exposure, and sensitivity to checkpoint blockade. Therefore, nutritional effects must be validated within specific cancer contexts. Fourth, host metabolic status is a major modifier. Obesity, diabetes, fatty liver disease, MASH, cachexia, sarcopenia, renal dysfunction, liver dysfunction, and steroid use may all change how a patient responds to dietary intervention. For example, fasting-like interventions may be unsafe in malnourished or cachectic patients. Ketogenic diets may be risky in patients with severe weight loss, liver disease, or uncontrolled diabetes. High-fiber interventions may worsen gastrointestinal symptoms in some patients. Artificial sweeteners may disrupt microbiota-dependent immune responses. Thus, safety cannot be separated from efficacy. Fifth, the microbiota introduces substantial interindividual variability. Baseline microbial composition, antibiotic exposure, proton pump inhibitor use, diet history, geography, and gastrointestinal toxicity may determine whether a nutritional intervention improves or impairs immune response. Without microbiome-aware study designs, dietary interventions may produce inconsistent results across cohorts. For these reasons, nutritional interventions should be viewed as precision immunometabolic strategies rather than universal anticancer diets. Future clinical trials should integrate dietary assessment, body composition analysis, metabolic biomarkers, microbiome profiling, immune monitoring, and treatment outcomes. Trials should also include nursing-relevant endpoints, such as adherence, tolerability, symptom burden, body weight change, sarcopenia risk, quality of life, and patient understanding of dietary recommendations. From a clinical perspective, the most appropriate current approach is not to recommend extreme diets broadly, but to identify nutritional risk, prevent harmful self-directed interventions, document major dietary exposures, and coordinate individualized care. Nutritional modulation of immunotherapy response is promising, but it must be translated through careful evidence generation, multidisciplinary management, and patient-centered nursing practice. Importantly, the mechanisms discussed in this review should not be interpreted as uniformly applicable to all cancer types. Current evidence is unevenly distributed across tumor models and clinical settings, with relatively more data from melanoma, colorectal cancer, breast cancer, hepatocellular carcinoma, renal cell carcinoma, lung cancer, and selected gastrointestinal tumors (15, 16, 21). Nutritional regulation of antitumor immunity is likely to differ according to tumor histology, anatomical site, baseline immune infiltration, stromal architecture, metabolic phenotype, microbiome dependence, treatment modality, and host nutritional status (187, 188). Therefore, this review uses “antitumor immunity” and “immunotherapy response” to describe shared immunometabolic principles, while emphasizing that cancer-type-specific validation is required before translating these findings into dietary recommendations.

### 10.4 Current guideline gap and evidence-based recommendations

A major limitation in current clinical practice is that few dietary recommendations for patients receiving cancer therapy are directly supported by cancer-therapy-specific guidelines. Existing evidence-based guidelines mainly support general cancer nutrition care, including routine malnutrition screening, assessment of weight loss and dietary intake, preservation of muscle mass, management of cachexia, individualized nutritional counseling, and multidisciplinary referral (189–191). In contrast, most immunometabolic dietary strategies discussed in this review, including fasting-mimicking diets, ketogenic diets, severe carbohydrate restriction, methionine or protein restriction, microbiome-directed nutrition, high-dose probiotics, artificial sweetener substitution, and supplement regimens, remain investigational and should not be recommended as routine anticancer or immunotherapy-enhancing interventions outside appropriate clinical evaluation. Cancer survivorship guidelines also emphasize healthy dietary patterns, weight management, physical activity, and cautious use of dietary supplements, but they do not provide immunotherapy-specific prescriptions for these experimental nutritional strategies (192–194). Therefore, the practical implication for oncology nursing is not to prescribe a specific anticancer diet, but to screen for malnutrition and unsafe dietary practices, document supplement and diet-product use, monitor symptoms and body composition, educate patients about the limits of evidence, and refer high-risk patients for dietitian- or physician-led management.

### 10.5 Evidence strength and clinical interpretation

The evidence reviewed here should be interpreted according to its level of clinical maturity (189, 195). Some areas, such as malnutrition screening, cachexia management, nutritional counseling, symptom monitoring, and multidisciplinary referral, are supported by clinical nutrition and oncology supportive-care guidelines (189, 191, 195). In contrast, many nutrition-based immunomodulatory strategies, including fasting-mimicking diets, ketogenic diets, severe carbohydrate restriction, amino acid restriction, microbiome-directed nutrition, high-dose probiotics, artificial sweetener substitution, and supplement regimens, are supported mainly by preclinical studies, observational cohorts, or early-phase clinical investigations (15, 196). These strategies should therefore be described as investigational rather than recommended clinical interventions. To avoid overinterpretation, this review uses mechanistic evidence to identify potential nursing-relevant assessment domains, not to establish definitive dietary prescriptions for patients receiving immunotherapy.

## 11 Nursing implications: from mechanistic evidence to safe clinical practice

The following section should be interpreted as nursing-relevant considerations rather than formal clinical practice recommendations. Current evidence does not support routine prescription of specific anticancer diets, fasting-mimicking diets, ketogenic diets, amino acid restriction, microbiome-directed nutrition, artificial sweetener substitution, or supplement regimens for all patients receiving immunotherapy. Instead, the nursing relevance of this evidence lies in risk identification, patient education, dietary behavior documentation, symptom monitoring, and timely multidisciplinary referral. Where guideline-based recommendations are available, they mainly concern malnutrition screening, nutritional assessment, cachexia management, symptom monitoring, and referral to nutrition professionals rather than immunotherapy-specific dietary prescriptions. The growing evidence linking nutrition, metabolism, the gut microbiota, and tumor immunity has direct implications for oncology nursing. To avoid repeating intervention-specific cautions across sections, this section integrates the nursing implications into a unified clinical framework for screening, education, monitoring, and multidisciplinary referral (197–199). Patients receiving immunotherapy are increasingly exposed to information about fasting, ketogenic diets, sugar restriction, artificial sweeteners, probiotics, high-fiber diets, amino acid restriction, and dietary supplements. Many of these strategies are discussed in scientific literature, social media, commercial nutrition programs, and patient communities, but the quality of evidence and clinical applicability vary widely (200–202). Therefore, nurses play a critical role in translating emerging mechanistic findings into safe, individualized, and patient-centered nutritional care. Nursing implications should not be understood as simple dietary instruction. Rather, oncology nursing can provide a structured clinical interface between experimental nutritional immunology and real-world patient behavior. Nurses are often the first to detect weight loss, reduced food intake, gastrointestinal symptoms, fatigue, supplement use, self-directed fasting, or misunderstanding of “anticancer diets.” This position allows nurses to identify nutritional risk early, prevent unsafe dietary practices, support adherence to therapy, and coordinate multidisciplinary care. In the context of immunotherapy, nutritional monitoring may also help interpret treatment tolerance, immune-related adverse events, metabolic complications, and potential modifiers of treatment response.

### 11.1 Nutritional screening before immunotherapy

Before immunotherapy begins, nutritional screening should be performed not only to identify malnutrition, but also to assess dietary behaviors that may influence immune-metabolic status (190, 191). Traditional screening focuses on body weight, recent weight loss, appetite, dietary intake, body mass index, and functional status (189, 203). These remain essential. However, the emerging field of nutritional immuno-oncology suggests that additional factors should be incorporated, including sarcopenia risk, metabolic disease, fatty liver disease, diabetes, gastrointestinal symptoms, artificial sweetener use, supplement intake, probiotic use, and extreme dietary practices (16, 190). Baseline assessment should include recent weight change, unintentional weight loss, muscle loss, appetite, oral intake, nausea, vomiting, diarrhea, constipation, dysphagia, taste changes, fatigue, and performance status. BMI should be interpreted cautiously because it does not distinguish adiposity from muscle mass. Patients with normal BMI may have sarcopenia, whereas patients with obesity may have sarcopenic obesity or metabolic dysfunction (204, 205). These conditions may influence treatment tolerance and immune competence. Assessment of muscle mass through imaging, physical function, grip strength, or clinical observation may be useful when available.

Metabolic comorbidities should also be evaluated. Diabetes, insulin resistance, dyslipidemia, fatty liver disease, metabolic dysfunction-associated steatohepatitis, liver cirrhosis, renal dysfunction, and steroid use may all modify the safety and biological effects of nutritional intervention (28, 190, 206). For example, fasting-mimicking diet has shown feasibility and systemic metabolic effects in patients with cancer, but this does not mean that fasting-like interventions are safe for every patient (15). Patients with cachexia, poor oral intake, diabetes, or advanced liver disease may be vulnerable to hypoglycemia, dehydration, muscle loss, or treatment intolerance. Dietary behavior assessment should include specific questions about sugar intake, sugar-free beverages, artificial sweeteners, ketogenic diets, intermittent fasting, low-protein diets, high-protein supplementation, fiber supplements, probiotics, herbal products, omega-3 products, and commercial anticancer diets. This is particularly important because sucralose consumption has been reported to impair immunotherapy response through microbiome disruption (17). Therefore, nurses should not only ask whether patients “eat sugar,” but also whether they use diet drinks, low-calorie sweeteners, sugar-free foods, or diabetic products. Equally important is assessing the patient’s understanding of nutrition and cancer. Some patients believe that all sugar must be eliminated, that fasting can starve cancer, that ketogenic diets are universally anticancer, or that natural supplements are always safe. Identifying these beliefs before treatment provides an opportunity for early education. Nutritional screening before immunotherapy should therefore combine clinical assessment, dietary history, metabolic risk evaluation, and patient belief assessment.

### 11.2 Patient education on extreme diets

Patient education is one of the most important nursing responsibilities in nutritional immuno-oncology. Many patients are motivated to improve treatment response through diet, but they may adopt extreme practices without professional guidance (190, 207). These practices may include prolonged fasting, strict ketogenic diets, severe sugar restriction, methionine or protein restriction, excessive fiber intake, high-dose probiotics, artificial sweetener substitution, plant-derived compound supplementation, or multiple commercial nutrition products (25, 188, 208). Nurses can support balanced patient education by acknowledging emerging scientific interest while clearly explaining that most diet-based immunotherapy-enhancing strategies remain investigational. Fasting-like interventions are a common source of confusion. Experimental studies suggest that fasting or fasting-mimicking diet may enhance immunotherapy response in some tumor models, including low-immunogenic breast cancer (21). However, this does not justify unsupervised fasting during cancer treatment. Patients with malnutrition, sarcopenia, cachexia, diabetes, liver dysfunction, renal dysfunction, infection, or treatment-related gastrointestinal symptoms may be harmed by caloric restriction. Nurses should explain that structured fasting-mimicking protocols studied in research settings are not equivalent to skipping meals or prolonged starvation.

Ketogenic diets require similar caution. Ketogenesis and β-hydroxybutyrate-related mechanisms may reduce immunosuppressive stromal signaling and improve checkpoint blockade response in selected models (22). However, strict ketogenic diets can reduce total calorie intake, fiber intake, and dietary variety. They may worsen constipation, fatigue, dyslipidemia, weight loss, or poor treatment tolerance. Patients should be advised not to start a strict ketogenic diet without discussion with the oncology team and a registered dietitian. Amino acid restriction also requires careful education. Methionine restriction may appear attractive as a tumor metabolism strategy, but methionine restriction-induced sulfur deficiency has been shown to impair antitumor immunity through gut microbiota-dependent mechanisms (25). This finding is especially important for patient counseling because some patients may attempt low-protein or amino acid-restricted diets to “starve” cancer. Nurses should emphasize that immune cells also require adequate amino acids, and excessive protein restriction may worsen muscle loss and immune dysfunction. High-fiber and microbiome-directed interventions should also be discussed carefully. Although dietary fiber is generally beneficial for bowel function and metabolic health, it does not consistently enhance immune checkpoint blockade efficacy across all experimental tumor models (35). Patients with diarrhea, bowel obstruction risk, abdominal pain, radiation enteritis, immune-related colitis, or severe bloating may not tolerate high-fiber strategies. Similarly, probiotics and fermented products should be considered in the context of immune status, gastrointestinal symptoms, infection risk, and clinical guidance.

Artificial sweeteners are particularly relevant to patient education. Patients trying to avoid sugar often increase consumption of sucralose-containing beverages or low-calorie foods. Because sucralose has been linked to impaired immunotherapy response through microbiome disruption, nurses should educate patients that sugar-free does not necessarily mean biologically neutral (17). This message should be delivered carefully, avoiding alarmism while encouraging patients to report sweetener use and avoid excessive reliance on artificial sweeteners during immunotherapy. Overall, patient education should avoid both extremes: it should not dismiss nutrition as irrelevant, but it should also not overstate experimental findings as clinical prescriptions. The safest message is that nutritional strategies should be individualized, supervised, and integrated with cancer treatment goals.

### 11.3 Monitoring during immunotherapy

Nutritional monitoring during immunotherapy should be continuous because dietary intake, symptoms, body composition, and metabolic status can change throughout treatment (190, 191). Monitoring should include both conventional nutritional parameters and diet-related behaviors that may influence immune-metabolic function (16). Body weight and food intake should be assessed regularly. Nurses should document unintentional weight loss, reduced appetite, early satiety, meal skipping, decreased protein intake, and changes in dietary pattern (189, 203). However, stable body weight does not always indicate nutritional stability. Patients may lose muscle while gaining or retaining fat, particularly in the setting of inactivity, inflammation, steroid exposure, or metabolic disease (204, 209). Therefore, fatigue, weakness, reduced mobility, and suspected sarcopenia should be monitored alongside weight.

Gastrointestinal symptoms require careful evaluation. Nausea, vomiting, diarrhea, constipation, bloating, abdominal pain, and appetite changes may be caused by treatment toxicity, immune-related adverse events, dietary changes, supplements, or infection. In patients receiving immune checkpoint inhibitors, distinguishing immune-related colitis from diet-related diarrhea is especially important. Nurses should ask about recent increases in fiber, probiotics, magnesium-containing supplements, artificial sweeteners, ketogenic products, or fasting practices when gastrointestinal symptoms occur (210, 211). Metabolic parameters should also be monitored when clinically indicated. Blood glucose is important in patients with diabetes, steroid exposure, fasting practices, or major carbohydrate restriction. Lipid profiles may be relevant for patients following ketogenic diets or taking high-fat supplements. Liver function tests should be interpreted in the context of immunotherapy, fatty liver disease, viral hepatitis, alcohol use, supplements, and metabolic syndrome. Renal function should be considered when patients use high-protein supplements, amino acid products, or dehydration-prone diets.

Nurses should also monitor use of non-prescribed products. Patients may take fish oil, omega-3 capsules, exogenous ketones, branched-chain amino acids, collagen, herbal extracts, sulforaphane products, probiotics, prebiotics, fiber powders, artificial sweeteners, or commercial anticancer supplements (196, 212). These products may affect gastrointestinal symptoms, bleeding risk, liver function, metabolic control, or treatment adherence (17, 190). Documentation is essential because patients may not consider supplements or diet products as “medications.” Self-directed dietary restriction should be actively screened (196, 213). Patients may fast before treatment, avoid carbohydrates, restrict protein, eliminate multiple food groups, or follow online anticancer diets. Nurses should ask about these behaviors in a nonjudgmental way. Open-ended questions such as “Have you made any major changes to your diet since starting treatment?” or “Are you avoiding any foods because of cancer?” may reveal practices that patients would otherwise not disclose (21, 208, 214).

Effective monitoring during immunotherapy therefore requires a broad view of nutrition. It should include intake, symptoms, metabolic status, body composition, supplement use, sweetener exposure, and patient behavior. This approach may help identify nutritional risk, detect potentially unsafe practices, and support more accurate interpretation of treatment-related symptoms (17, 188).

### 11.4 Multidisciplinary nutrition management

Nutritional care during immunotherapy is best viewed as a multidisciplinary process, particularly when patients have malnutrition, cachexia, metabolic comorbidities, gastrointestinal toxicity, or self-directed use of restrictive diets or supplements (215). Nurses should not be expected to independently prescribe complex dietary interventions such as fasting-mimicking diet, ketogenic diet, microbiome-directed nutrition, amino acid restriction, or supplement regimens (189, 206). Instead, nurses play a central coordinating role by identifying risk, educating patients, documenting behaviors, monitoring symptoms, and referring patients to dietitians, oncologists, pharmacists, endocrinologists, hepatologists, or other specialists when needed. A useful model is nurse-led nutritional surveillance during immunotherapy (189, 190). This model does not mean that nurses replace dietitians. Rather, nurses provide systematic, repeated, clinically embedded assessment throughout the treatment course. Nurse-led surveillance could include baseline nutritional screening, structured dietary behavior questions, symptom-triggered reassessment, documentation of extreme diets and supplement use, monitoring of body weight and functional decline, and referral pathways for high-risk patients (196, 206, 213). This approach is particularly valuable because immunotherapy is often administered over long periods, during which patient dietary behavior may change.

Multidisciplinary care is especially important for high-risk groups. Patients with cachexia, sarcopenia, severe weight loss, diabetes, fatty liver disease, liver cirrhosis, renal dysfunction, gastrointestinal toxicity, or poor performance status require individualized nutritional planning. Patients considering fasting, ketogenic diets, protein restriction, high-dose supplements, or major dietary changes should be referred for professional evaluation (15, 25, 216). Pharmacists may help assess supplement-drug interactions, bleeding risk, hepatotoxicity, and metabolic complications. Dietitians can provide practical dietary plans that preserve nutritional adequacy while respecting patient preferences and treatment goals (189). Nursing communication should also support shared decision-making. Patients often pursue dietary changes because they want control over their disease and treatment. Dismissing these concerns may reduce trust. A more effective approach is to acknowledge the patient’s motivation, explain the evidence level, identify safety concerns, and offer supervised alternatives (189, 196, 213). For example, instead of simply saying that fasting is not allowed, nurses can explain that fasting-like interventions are being studied but may be unsafe in patients with weight loss or diabetes, and that nutritional optimization should be coordinated with the care team. In this way, oncology nursing may help translate mechanistic nutritional research into safer, patient-centered monitoring and referral pathways, without implying that experimental dietary strategies are ready for routine clinical prescription. The nursing contribution lies in early recognition, practical education, behavioral monitoring, symptom interpretation, and multidisciplinary coordination. These roles are essential if nutritional immuno-oncology is to move from experimental models toward patient-centered cancer care. To make nursing-led nutritional surveillance more feasible and reproducible, validated instruments should be incorporated into routine assessment and prospective immunotherapy cohorts. For nutritional risk screening, nurses may use brief validated tools such as the Malnutrition Screening Tool, Malnutrition Universal Screening Tool, Nutritional Risk Screening 2002, Mini Nutritional Assessment, NUTRISCORE, or the Patient-Generated Subjective Global Assessment Short Form, depending on the clinical setting and local workflow (191, 217). For more comprehensive nutritional assessment, the scored Patient-Generated Subjective Global Assessment is particularly useful in oncology because it captures weight loss, food intake, nutrition-impact symptoms, functional status, disease burden, metabolic demand, and physical examination findings (218). To capture dietary intake more directly, repeated 24-hour dietary recalls, food diaries, food frequency questionnaires, and web-based tools such as the Automated Self-Administered 24-Hour Dietary Assessment Tool may be used in clinical studies or structured follow-up programs (219–221). These tools can be paired with patient-reported outcome instruments, such as PRO-CTCAE for symptomatic adverse events and EORTC QLQ-C30 for quality of life, to examine associations between dietary behavior, symptom burden, treatment tolerance, and clinical outcomes (222, 223). In the context of nutritional immuno-oncology, additional structured questions should document supplement use, probiotics, prebiotics, artificial sweeteners, fasting behavior, ketogenic or low-carbohydrate diets, protein restriction, and major dietary exclusions, because these exposures are not consistently captured by standard nutritional screening tools. To translate mechanistic evidence into clinical practice, **Table 3** outlines nursing-relevant assessment domains, biological rationales, and practical actions for nutritional management during cancer immunotherapy. This framework emphasizes that oncology nurses can contribute to precision nutritional care through early risk identification, patient education, dietary behavior monitoring, and multidisciplinary coordination.

**Table 3:** Nursing implications for nutritional management during cancer immunotherapy.

Nursing domain	What nurses should assess	Mechanistic rationale	Practical nursing actions	Related evidence
Baseline nutritional screening	Recent weight loss, BMI, appetite, dietary intake, performance status, fatigue	Malnutrition and reduced intake may impair immune competence and treatment tolerance	Screen before immunotherapy and repeat during treatment cycles	FMD safety and metabolic remodeling: PMID 34789537
Sarcopenia and body composition	Muscle loss, weakness, reduced mobility, sarcopenic obesity	BMI alone may miss muscle depletion and metabolic risk	Monitor functional decline and refer early to dietitians or rehabilitation teams	Amino acid and leucine-related immune effects: PMID 38447573
Metabolic comorbidities	Diabetes, dyslipidemia, fatty liver, MASH, insulin resistance, liver enzymes	Host metabolic disease can reshape TIME and alter ICB response	Document metabolic history and coordinate with physicians for high-risk patients	MASH-HCC/SQLE/anti-PD-1: PMID 38744443
Fasting or FMD behavior	Meal skipping, intermittent fasting, prolonged fasting, commercial fasting programs	Fasting-like interventions may alter systemic metabolism and immunity but can be unsafe in vulnerable patients	Explain that FMD is structured and supervised, not self-directed starvation	PMID 34789537; PMID 36001966; PMID 39879585
Ketogenic diet use	Strict carbohydrate restriction, high-fat intake, exogenous ketones, MCT oil	Ketone bodies may modulate CAF signaling and ICB response, but restrictive diets may worsen malnutrition	Assess weight, bowel function, lipid profile, glucose control, and dietitian involvement	PMID 35247887; PMID 37609341
Carbohydrate beliefs	“Sugar feeds cancer” beliefs, low-sugar diets, high-sugar drinks, fruit avoidance	Carbohydrate-derived immune effects are context dependent and may involve microbiota, adipocytes, or hepatocytes	Provide balanced education and avoid simplistic anti-sugar messaging	PMID 37863051; PMID 37804509; PMID 40781141
Artificial sweetener exposure	Diet drinks, sugar-free foods, sucralose-containing products, diabetic products	Sucralose may disrupt microbiota and reduce immunotherapy response	Ask specifically about sweeteners, not only sugar intake	PMID 40742298
Protein and amino acid restriction	Low-protein diets, methionine restriction, avoidance of animal protein	Excessive amino acid restriction may impair immune fitness and worsen sarcopenia	Discourage unsupervised protein restriction and monitor muscle loss	PMID 37537369
High-protein or amino acid supplementation	Protein powders, BCAA, leucine, collagen, nutrition shakes	Amino acids may support muscle and immune-cell function, but supplements require context	Document supplement use and assess renal/liver function when clinically relevant	PMID 38447573
Fiber, probiotics, and microbiome-directed diets	Fiber powders, probiotics, fermented foods, prebiotics, antibiotics	Microbiota affects ICB response, but fiber benefit is not universal	Assess GI tolerance, diarrhea, bloating, antibiotic exposure, and immune-related colitis symptoms	PMID 36138151; PMID 40540354
Gastrointestinal symptom monitoring	Diarrhea, constipation, bloating, nausea, abdominal pain, appetite change	Symptoms may reflect diet changes, supplements, infection, or immune-related toxicity	Distinguish diet-related symptoms from immune-related colitis and escalate when needed	Microbiota and diet-related studies: PMID 36138151; PMID 40742298
Lipid-related assessment	Fish oil, omega-3, high-fat diets, dyslipidemia, bleeding risk, liver disease	Lipid mediators and inflammation-resolution pathways may influence immunotherapy response	Document lipid supplements and coordinate around procedures or anticoagulant use	PMID 38330013
Supplement and herbal product use	Sulforaphane products, herbal extracts, commercial anticancer supplements	Bioactive compounds may affect immune pathways but clinical safety varies	Encourage disclosure and coordinate with pharmacist/oncologist	PMID 38296593
Patient education	Understanding of anticancer diets, online advice, fear of sugar, supplement beliefs	Misinterpretation of mechanistic studies may lead to unsafe dietary practices	Use nonjudgmental counseling and explain evidence boundaries	Negative/context-dependent evidence: PMID 37537369; PMID 40540354; PMID 40742298
Nurse-led nutritional surveillance	Repeated dietary review, symptom-linked diet logs, supplement tracking, referral triggers	Nutritional behavior changes during long-term immunotherapy may influence safety and response	Establish structured monitoring during ICB treatment	Supports precision nutrition implementation across studies

## 12 Future perspectives

The field of nutritional regulation of the TIME is still evolving. Current evidence shows that nutrition can influence systemic metabolism, gut microbiota, immune-cell function, stromal barriers, and immunotherapy response. However, many findings remain preclinical, context-specific, or mechanistically incomplete. Future research should move beyond isolated nutrient effects and toward integrated models that capture the complexity of human diet, host metabolism, microbial ecology, tumor biology, and clinical care.

### 12.1 From single nutrients to nutritional ecosystems

Future studies should not only ask whether one nutrient is beneficial or harmful. Instead, nutrition should be studied as an ecosystem. Real-world diets are composed of multiple interacting components, including carbohydrates, proteins, fats, fiber, micronutrients, additives, fermented foods, supplements, and meal timing patterns (16, 188, 224). These components interact with the gut microbiota, host metabolic organs, immune cells, and cancer therapies. Therefore, the biological effect of a nutrient may depend on the broader dietary background. For example, the effect of dietary fiber may depend on baseline microbiota composition, fiber type, fermentation capacity, bowel function, and treatment regimen. The effect of carbohydrate intake may differ according to glucose control, adipose tissue function, liver metabolism, and artificial sweetener use. The effect of fasting-like interventions may depend on body composition, tumor type, treatment timing, and nutritional reserve (16, 188). The effect of amino acid restriction may depend on microbial sulfur metabolism and immune-cell requirements. These examples demonstrate that nutritional immunology cannot be reduced to a single nutrient-response model (15, 21). Future research should therefore integrate dietary pattern assessment, metabolomics, microbiome sequencing, immune profiling, and clinical outcomes. Longitudinal designs will be especially important because diet, microbiota, and immune states change over time. Studies should assess not only baseline diet, but also dietary changes during immunotherapy, antibiotic exposure, gastrointestinal toxicity, weight loss, supplement use, and adherence to nutritional interventions. Such approaches may help identify dynamic nutritional ecosystems that promote or impair immunotherapy response.

### 12.2 Biomarker-guided nutritional immunotherapy

Biomarker-guided nutritional immunotherapy is a promising future direction. Because nutritional interventions are context dependent, biomarkers are needed to identify which patients are likely to benefit, which patients may be harmed, and which mechanisms are active in a given clinical setting (16, 188). These biomarkers should include microbial, metabolic, immune, and body composition indicators. Gut microbiota profiling may help identify microbial communities associated with favorable or unfavorable response to nutritional intervention. Fecal microbiome structure, microbial diversity, functional gene pathways, and microbial metabolite-producing capacity may be more informative than taxonomic abundance alone. In addition, stool metabolomics may help identify microbial-derived mediators that connect diet with immune response (224, 225).

Serum and plasma metabolomics could provide complementary information. Candidate metabolites include β-hydroxybutyrate, TMAO, hydrogen sulfide-related metabolites, FAD-related pathways, short-chain fatty acids, bile acids, cholesterol metabolites, squalene pathway intermediates, amino acid metabolites, and lipid mediators derived from omega-3 fatty acids. These metabolites may help determine whether a dietary intervention has successfully induced the intended biological state. Immune biomarkers are also essential. CD8^+^ T-cell infiltration, T-cell exhaustion markers, cytokine profiles, antigen presentation signatures, MDSC abundance, TAM phenotypes, neutrophil states, and Treg frequency may help connect nutritional intervention with immune outcome (188, 226). Spatial profiling may be particularly valuable because nutritional interventions may affect not only immune-cell abundance but also immune-cell localization (227–229). For example, weakening CAF-mediated barriers may allow T cells to move from stromal regions into tumor nests. Body composition and metabolic biomarkers should be included as well. Sarcopenia, sarcopenic obesity, visceral adiposity, fatty liver disease, MASH, insulin resistance, dyslipidemia, and systemic inflammation may strongly influence the safety and efficacy of nutritional strategies. Imaging-based muscle and fat assessment, liver fat evaluation, inflammatory markers, glucose control, and lipid profiles could help stratify patients before dietary intervention (189, 204, 205).

Ultimately, biomarker-guided nutritional immunotherapy should aim to match the right intervention to the right patient at the right time. Fasting-like strategies may be appropriate only for patients with adequate nutritional reserve. Ketogenic approaches may require metabolic and hepatic screening. Microbiome-directed strategies may require baseline microbial assessment. Amino acid or lipid interventions may require metabolomic and immune monitoring. This precision approach is necessary to avoid unsafe generalized dietary recommendations.

### 12.3 Nursing-led clinical trials

Nursing-led and nursing-participatory clinical trials represent an important future direction. Many nutritional immunotherapy studies focus on biological endpoints but underemphasize patient behavior, adherence, symptom burden, safety, and education. Nurses are uniquely positioned to address these gaps because they interact closely with patients throughout treatment and routinely monitor symptoms, diet-related behaviors, and quality of life (190, 206, 230). Future nursing-led studies could evaluate structured nutritional surveillance during immunotherapy. Such studies might include baseline nutritional risk screening, repeated dietary intake assessment, documentation of artificial sweetener use, tracking of supplement and probiotic consumption, monitoring of fasting or ketogenic behaviors, and early referral for high-risk patients (17, 188, 191). Outcomes could include treatment adherence, immune-related adverse events, nutritional deterioration, sarcopenia progression, patient knowledge, quality of life, and clinical response.

Another important direction is patient education intervention. Nurses could lead trials testing whether structured education reduces unsafe dietary practices, improves reporting of supplement use, prevents excessive restriction, or improves nutritional adequacy during immunotherapy (213, 230). Education modules could address common misconceptions, such as “sugar feeds cancer,” “fasting is always beneficial,” “ketogenic diets are universally anticancer,” “natural supplements are always safe,” or “sugar-free products are automatically healthier.” Nursing research could also contribute to microbiome and dietary studies by improving the accuracy of real-world dietary data (15, 17, 216). Food frequency questionnaires, diet diaries, mobile app-based dietary recording, symptom-linked food logs, and sweetener exposure assessments could be incorporated into immunotherapy cohorts. Nurses could help collect longitudinal data that reflect actual patient behavior rather than idealized dietary protocols (16, 188, 220). Body composition monitoring is another promising nursing-related area. Prospective studies could evaluate whether routine monitoring of weight change, muscle loss, fatigue, physical function, and nutritional intake improves early detection of sarcopenia or cachexia during immunotherapy. Such trials would be especially valuable in patients with obesity, because sarcopenic obesity may be missed when BMI is used alone. Finally, nursing-led clinical trials should emphasize feasibility and equity. Nutritional interventions must be practical, culturally adaptable, affordable, and acceptable to patients with different socioeconomic backgrounds. A diet that is biologically promising but difficult to follow may not be clinically useful. Nurses can help design interventions that align with patient preferences, symptoms, family support, food access, and treatment schedules (230, 231).

In conclusion, future nutritional immuno-oncology should combine mechanistic rigor with clinical realism. The field should move from isolated nutrients to nutritional ecosystems, from population-level assumptions to biomarker-guided strategies, and from experimental protocols to patient-centered implementation. Oncology nursing will be essential in this transition because nurses can connect biological evidence with safe, individualized, and sustainable care during immunotherapy.

## 13 Conclusion

Nutrition is emerging as an important regulator of the tumor immune microenvironment rather than a passive supportive-care factor. Accumulating experimental and translational evidence indicates that dietary inputs, fasting-like interventions, ketone bodies, carbohydrate-derived signals, amino acid availability, lipid mediators, artificial sweeteners, and microbiota-derived metabolites can reshape antitumor immunity through multiple interconnected mechanisms. These mechanisms involve systemic nutrient availability, gut microbial ecology, adipose tissue remodeling, hepatocyte metabolic signaling, CAF-mediated stromal barriers, myeloid-cell reprogramming, CD8^+^ T-cell metabolic fitness, and immune checkpoint blockade responsiveness. A key message from this field is that nutritional regulation of cancer immunity is highly context dependent. The same nutritional factor may enhance antitumor immunity in one tumor model but have neutral or harmful effects in another. Fasting-mimicking diet, ketogenic interventions, omega-3-related lipid pathways, leucine, sulforaphane, TMAO, galactose, and microbial metabolites may improve immunotherapy response under specific conditions. Conversely, methionine restriction-induced sulfur deficiency, excessive artificial sweetener exposure, or nonspecific dietary fiber supplementation may impair or fail to improve immune checkpoint blockade in certain contexts. Therefore, nutrition should not be framed as a universal anticancer prescription, but as a precision immunometabolic variable that requires careful evaluation. For clinical practice, these findings have important nursing implications. Patients receiving immunotherapy frequently seek dietary strategies to improve treatment response, but unsupervised fasting, strict ketogenic diets, protein restriction, excessive supplement use, artificial sweetener substitution, and microbiome-directed self-interventions may create nutritional and metabolic risks. Oncology nurses are uniquely positioned to identify these behaviors, assess nutritional risk, monitor weight and muscle loss, evaluate gastrointestinal symptoms, document supplement and sweetener use, and coordinate multidisciplinary nutrition care. Nurse-led nutritional surveillance may become an important component of safe immunotherapy management. Future research should move beyond single-nutrient models and toward integrated nutritional ecosystems that include diet patterns, gut microbiota, serum metabolomics, immune-cell states, body composition, metabolic comorbidities, and treatment outcomes. Biomarker-guided trials are needed to determine which patients may benefit from specific nutritional interventions and which patients may be harmed. Ultimately, the goal is not to replace anticancer therapy with diet, but to understand how nutrition can be safely and precisely used to support antitumor immunity, improve immunotherapy response, and enhance patient-centered cancer care.
